# Dissecting the Biochemical and Transcriptomic Effects of a Locally Applied Heat Treatment on Developing Cabernet Sauvignon Grape Berries

**DOI:** 10.3389/fpls.2017.00053

**Published:** 2017-01-31

**Authors:** Fatma Lecourieux, Christian Kappel, Philippe Pieri, Justine Charon, Jérémy Pillet, Ghislaine Hilbert, Christel Renaud, Eric Gomès, Serge Delrot, David Lecourieux

**Affiliations:** ^1^Centre National de la Recherche Scientifique, Institut des Sciences de la Vigne et du Vin, UMR Ecophysiologie et Génomique Fonctionnelle de la VigneVillenave d'Ornon, France; ^2^Institut National de la Recherche Agronomique (INRA), Institut des Sciences de la Vigne et du Vin, UMR Ecophysiologie et Génomique Fonctionnelle de la VigneVillenave d'Ornon, France; ^3^Université de Bordeaux, Institut des Sciences de la Vigne et du Vin, UMR Ecophysiologie et Génomique Fonctionnelle de la VigneVillenave d'Ornon, France

**Keywords:** grapevine, berry development, microclimate, high temperature, microarrays, metabolomics/metabolite profiling, climate change

## Abstract

Reproductive development of grapevine and berry composition are both strongly influenced by temperature. To date, the molecular mechanisms involved in grapevine berries response to high temperatures are poorly understood. Unlike recent data that addressed the effects on berry development of elevated temperatures applied at the whole plant level, the present work particularly focuses on the fruit responses triggered by direct exposure to heat treatment (HT). In the context of climate change, this work focusing on temperature effect at the microclimate level is of particular interest as it can help to better understand the consequences of leaf removal (a common viticultural practice) on berry development. HT (+ 8°C) was locally applied to clusters from Cabernet Sauvignon fruiting cuttings at three different developmental stages (middle green, veraison and middle ripening). Samples were collected 1, 7, and 14 days after treatment and used for metabolic and transcriptomic analyses. The results showed dramatic and specific biochemical and transcriptomic changes in heat exposed berries, depending on the developmental stage and the stress duration. When applied at the herbaceous stage, HT delayed the onset of veraison. Heating also strongly altered the berry concentration of amino acids and organic acids (e.g., phenylalanine, γ-aminobutyric acid and malate) and decreased the anthocyanin content at maturity. These physiological alterations could be partly explained by the deep remodeling of transcriptome in heated berries. More than 7000 genes were deregulated in at least one of the nine experimental conditions. The most affected processes belong to the categories “stress responses,” “protein metabolism” and “secondary metabolism,” highlighting the intrinsic capacity of grape berries to perceive HT and to build adaptive responses. Additionally, important changes in processes related to “transport,” “hormone” and “cell wall” might contribute to the postponing of veraison. Finally, opposite effects depending on heating duration were observed for genes encoding enzymes of the general phenylpropanoid pathway, suggesting that the HT-induced decrease in anthocyanin content may result from a combination of transcript abundance and product degradation.

## Introduction

Grapevine is probably the fruit species whose economical value depends the most on climatic conditions. Besides the soil and local viticultural practices, which are components of the so-called “terroir” effect, grapevine and wine quality are determined by vintage that integrates the different climatic parameters experienced by the plant during growth and ripening. Among these environmental factors, temperature is a major regulator affecting both grapevine phenology and fruit composition (Schultz, 2000; Jones et al., 2005).

The Intergovernmental Panel on Climate Change (IPCC) predicts a significant increase of mean temperatures ranging between 1.8 and 4°C by the end of the 21st century, depending on the scenario (IPCC, 2013). Given that grapevine varieties have been ranked according to their thermal preferences (Huglin, 1978), this may lead to change the varieties traditionally grown in AOC areas, or even threaten the sustainability of some Mediterranean viticultural areas (Hannah et al., 2013; Fraga et al., 2016).

However, mean temperature perceived by the plant may be altered by common viticultural practices that may be envisaged as a mean to cope with the effects of climate change (Van Leeuwen et al., 2013). For example, the extent of leaf removal directly affects light exposure and temperature of the berries. Berry temperatures depend on row orientation and significantly differ between east and west-exposed sides in the case of north-south row orientation. The temperature also interacts with the leaf/fruit (source/sink) relationships to determine yield (Sadras and Moran, 2013) and with the circadian clock to determine transcriptomic responses (Rienth et al., 2014). Moderate increases of berry temperatures obtained in open-top chambers uncouple the anthocyanin and sugar contents in red varieties (Sadras and Moran, 2012) and affect berry sensory traits (Sadras et al., 2013). Because of the direct irradiative effects, the temperature of reproductive organs may differ very significantly from that of the whole plant or surrounding air (Spayd et al., 2002; Pieri and Fermaud, 2005; Cola et al., 2009).

Secondary metabolites, which are among the most prominent discriminating compounds for berry quality, are synthesized *in situ* under the direct influence of the local micro-environment (mostly temperature and light conditions). In this context, it is important to investigate the effects of high temperatures on berry metabolism and composition. These effects depend on the imposed temperature gradient, and on the time and duration of the heat stress. During flowering, high temperatures inhibit berry set and reduce yield (Greer and Weston, 2010). After fruit set, high temperatures stimulate sugar accumulation at the expense of other qualitative compounds (Greer and Weston, 2010) such as organic acids (Champagnol, 1984), flavonols, anthocyanins, amino acids (Spayd et al., 2002; Pereira et al., 2006; Mori et al., 2007; Tarara et al., 2008; Cohen et al., 2012) and aromas (Schultz, 2000). Together with other parameters such as the carbohydrate source-sink ratio (Ollat and Gaudillère, 1998; Parker et al., 2014; de Toda and Balda, 2015), ambient air temperature is a well-known factor that influences berry growth and veraison timing (Duchene et al., 2010), but the impact of heat treatment (HT) at the berry level on ripening onset has not been described extensively.

In the present work, we compare the effects of HT (+8°C, 14 days) imposed on berry clusters at 3 different stages (green, veraison, ripening) of berry development. This experimental setup avoids the complexity of the responses involving both light and heat effects, and allows a detailed study of the relevance of developmental stages in the response to HT. Biochemical analysis and gene expression studies were conducted at 3 time points (1, 7, and 14 days) after the beginning of HT, which also allowed us to study the time course of the responses. Our results showed dramatic and specific biochemical and transcriptomic changes in heat exposed berries, depending on the developmental stage and the stress duration. Heating strongly altered the berry concentration of amino acids and organic acids and decreased the anthocyanin content at maturity. When applied at the herbaceous stage, HT delayed the onset of veraison. More than 7000 genes were deregulated in at least one of the nine experimental conditions contributing to the postponing of veraison. Finally, opposite effects depending on heating duration were observed for genes encoding enzymes of the general phenylpropanoid pathway, suggesting that the HT-induced decrease in anthocyanin content may result from a combination of transcript abundance and metabolite degradation.

## Materials and methods

### Plant material

Fruiting cuttings of *Vitis vinifera* L. cv Cabernet Sauvignon (Ollat and Gaudillère, 1998) were grown in a greenhouse, in 0.5 L pots containing a mixture of perlite, sand and vermiculite (1:1:1). A drip irrigation system supplied water and a complete nutrient solution to the roots five times a day all along the experiment, avoiding any water or nutrient shortage. All fruiting cuttings bore only one single cluster and lateral shoots were removed as soon as they appeared during growth. Before the experiment, the tip of each shoot was removed as soon as 16 leaves per plant were produced to maintain approximately the same leaf area in all plants and a high leaf to fruit ratio (Ollat and Gaudillère, 1998). Therefore, all bunches were assumed to experience neither water nor assimilate limitation. In addition, the fruit cuttings were selected on the basis of similar vegetative growth and vigor as well as size and compactness of bunches.

### Heat treatment, temperature measurements, and sampling

Both control and heat treatments were applied for 14 d periods, at 3 phenological stages, namely middle-green (30 days after fruit set), veraison (50% of berries turning to a visible red color) and middle-ripening (80 days after fruit set). Total soluble solids (TSS) content in berries was determined before, during and after each treatment using a digital refractometer (Atago, Tokyo). Before treatments, middle-green, veraison and middle-ripening berries displayed a TSS of 3.9 ± 0.3, 8.9 ± 1.4 and 14.1 ± 1.1°Bx, respectively (mean of replicates ± SD). Three sets of 25 fruiting cuttings were used as controls [GC (Green Control), VC (Veraison Control) and RC (Ripening Control)]; the temperature of their bunch closely followed greenhouse ambient air temperature. Three sets of 25 bunches from other fruiting cuttings were submitted to heat treatment: GHT (Green Heat Treatment), VHT (Veraison Heat Treatment) and RHT (Ripening Heat Treatment). The clusters were submitted to an elevated temperature airflow produced by fan heaters (common domestic models, used at 1000 W). Only bunches were heated since shoots, leaves and roots were all protected from the heated airflow by extruded polystyrene foam deflectors. Air heating was applied repeatedly during 14 d, from 7:00 a.m. to 7:00 p.m. every day to mimic the usual diurnal temperature course of exposed berries. To avoid any differential effect linked to airflow and possible mechanical stress, simple fan blowers were used to create a continuous airflow around the control clusters during 14 d, from 7:00 a.m. to 7:00 p.m. every day. The flesh temperatures in control and heated berries (10 replicates for each treatment) were monitored continuously by copper-constantan thermocouples inserted into the berries, connected to a Campbell datalogger (Campbell Scientific). After treatments, each set of fruiting cuttings was replaced in control conditions until harvest.

The experiment was conducted over 3 years. The samples collected the first year were used for biochemical and transcriptomic analysis. Samples from years 2 and 3 were used for biochemical and RT-qPCR analysis.

### Sampling

In order to analyse short- and long-term responses for each treatment, 3 control and 3 heat-stressed berries per cluster (25 clusters per condition) were sampled in the evening (7:00 p.m.) at 3 different time points after treatment (1, 7, and 14 d), immediately frozen in liquid nitrogen and stored at −80°C. To obtain 3 experimental replicates, each of the 3 berries collected per cluster was used to constitute 3 independent groups of 25 berries for each condition. The pool of deseeded berries from each group was used as a biological replication and underwent independent RNA extractions.

For the biochemical analysis conducted around harvest, 3 biological replicates were prepared as described above, with berries collected from the same 25 fruiting cutting used for each treatment. Then, frozen berries (5 berries per replicate) were slightly thawed and separated quickly into skin, pulp, and seed. The skin and pulp were immediately ground into fine powder in liquid nitrogen using mortar and pestle.

### Metabolites quantification

#### Sugars, organic acids, and amino acids contents

An aliquot of 500 mg fine powder of pulp was extracted sequentially with ethanol (80 and 50%), dried in a Speed-Vac, and re-dissolved in 2.5 mL de-ionized water. Glucose and fructose content were measured enzymatically with an automated micro-plate reader (Elx800UV, Biotek Instruments Inc., Winooski, VT, USA) according to the method of Gomez et al. (2007). Tartaric acid content was assessed by using the colorimetric method based on ammonium vanadate reactions (Pereira et al., 2006). Malic acid was determined using an enzyme-coupled spectrophotometric method that measures the change in absorbance at 340 nm from the reduction of NAD+ to NADH (Pereira et al., 2006). The amino acid content was determined after derivatization (Cohen and Michaud, 1993) using a Waters 2695 HPLC system equipped with Waters 474 fluorescence detector (Waters, Milford, MA, USA). Twenty amino acids were identified and quantified as described by Pereira et al. (2006). The results were expressed in μmol.L^−1^ juice.

#### Anthocyanins quantification

An aliquot of 500 mg of berry skin powder was freeze-dried for 72 h and the dried powder (50 mg) was extracted in 1.0 mL methanol containing 0.1% HCl (v/v). Extracts were filtered through a 0.45 μm polypropylene syringe filter (Pall Gelman Corp., Ann Harbor, MI, USA) for HPLC analysis. Each individual anthocyanin was analyzed with HPLC as described in Soubeyrand et al. (2014). Quantification was carried out by peak area integration at 520 nm. The concentration of individual anthocyanins was calculated in milligrams per gram (mg. g^−1^) of dry skin weight (DW) using malvidin 3-*O*-glucoside (Extrasynthese, Genay, France) as external standard.

### RNA extraction and cDNA production

Berries collected from Cabernet Sauvignon fruit cuttings were quickly frozen in liquid nitrogen, ground to a fine powder with a Dangoumau blender, and stored at −80°C prior to use. Total RNA from deseeded berries was extracted according to Lecourieux et al. (2010). RNA isolation was followed by DNase I treatment. The purity and quantity of the RNA were determined using a Nanodrop 1000 spectrophotometer (Thermo Scientific). RNA integrity was determined using a Bioanalyzer 2100 (Agilent) with RNA 6000 Nano Kit I (Agilent). For each sample, reverse transcription was performed from 2 μg of purified RNA using the Moloney murine leukemia virus reverse transcriptase (Promega) according to the manufacturer's instructions. The cDNA obtained was diluted (1:10) in distilled water.

### Microarray hybridization and data processing

Total RNAs extracted from 3 biological replicates per condition were hybridized with 60-mer oligoarrays bearing a set of probes for 29,582 unigenes (NimbleGen Gene Expression 12 × 135K Arrays). Labeling, hybridization and scanning were carried out at the GeT-transcriptomic platform (GenoToul-Toulouse, France). Microarray data were analyzed using the R and R/Bioconductor software (Gentleman et al., 2004; R Core Team, 2013). Quality control was done using the arrayQualityMetrics package (Kauffmann et al., 2009), one identified outlier (GHS14_1) was excluded from further analyses. Data were normalized using the Robust Multi-array Average algorithm (RMA) (Irizarry et al., 2003). Principal component analysis was done using the prcomp function, visualizations were made using ggplot2 (Wickham, 2009). Differentially expressed genes between treatment and control samples for all stages and treatment durations were identified using Limma package (Smyth, 2005). Differentials with absolute fold changes above 2 and BH (Benjamini and Hochberg, 1995) corrected *P*-values below 0.05 were considered significant. Significantly affected gene categories based on the MapMan Ontology (Thimm et al., 2004; Usadel et al., 2005) were identified using a Chi-square test. MapMan mappings for the Cribi 12X grapevine genome were based on closest homologs regarding to the *Arabidopsis thaliana* genome. Empirical cumulative distribution function plots for selected categories were made using the latticeExtra package. Gene annotations were taken from Grimplet et al. (2012).

The raw microarray data were submitted to Gene Expression Omnibus (NCBI) and are accessible through GEO accession number GSE86551.

## Results

### Heat treatment and berry temperature recording

**Figure 1:**
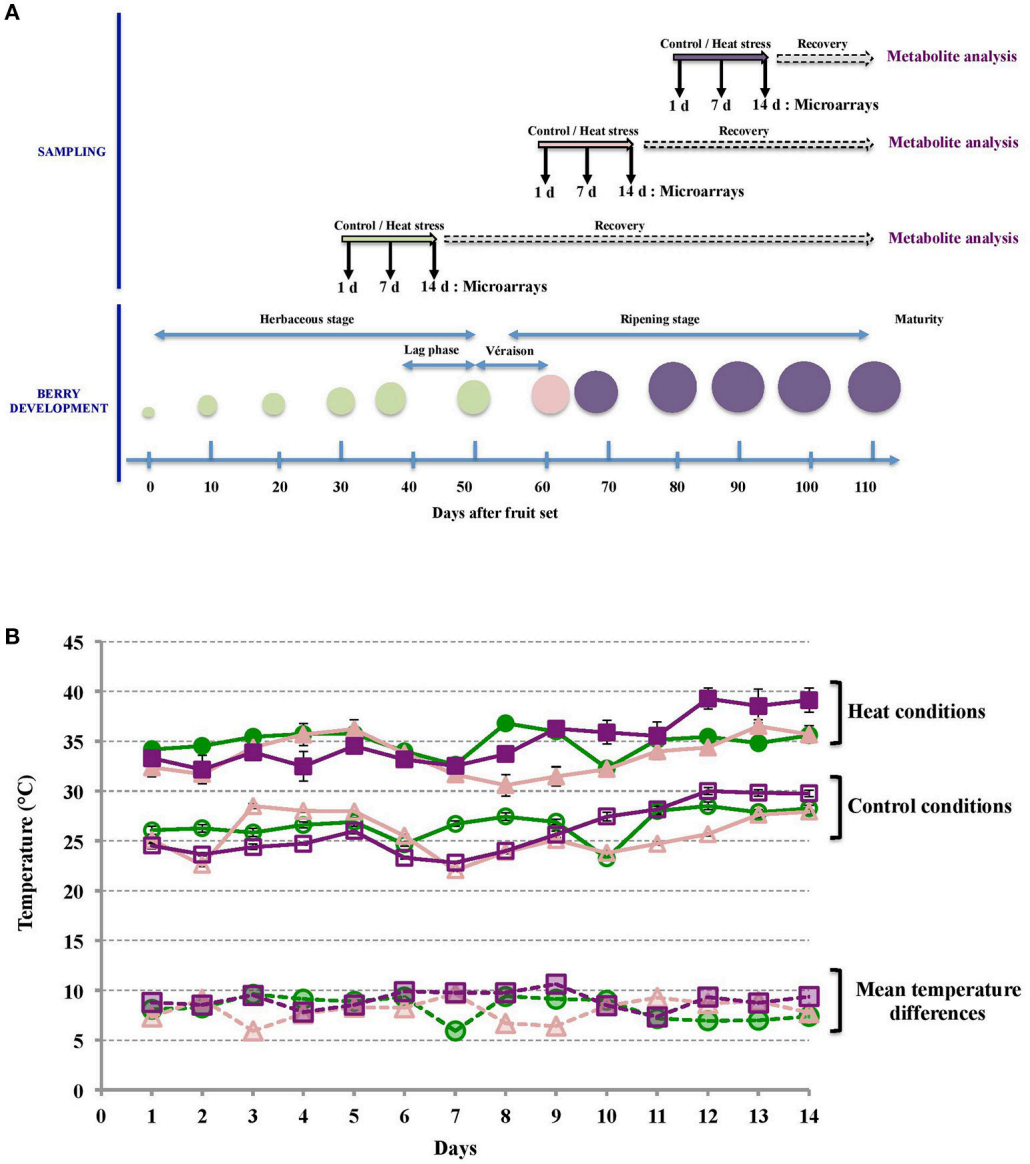
**Sampling and fruit temperature recording during treatments. (A)** Both control and HT were applied for 14 d periods, at middle-green, veraison (50% of berries turning to a visible red color) and middle-ripening. For transcriptomic analysis, samples were collected after 1, 7, and 14 d of treatment. For biochemical analysis, berries from each treatment condition were sampled at harvest. **(B)** Mean temperatures in control (opened symbols) and heated berries (filled symbols) during the herbaceous (green circles), veraison (pink triangles) and ripening (purple squares) periods were determined for each day of treatment. Average temperature differences between heated and control berries for each stage are showed (dashed lines).

HT was applied locally on clusters of Cabernet Sauvignon fruiting cuttings at 3 developmental stages, namely middle-green, veraison and middle-ripening (Figure 1A). Three different sets of 25 plants per condition (control or HT) were used for each developmental stage. HT was applied every day during 14 d from 7:00 a.m. to 7:00 p.m. to mimic usual daytime temperature course of sunlight-exposed berries. Under control conditions, average daytime berry flesh temperature measured were 26.5°C ± 1.4 (control green stage, GC clusters), 25.8°C ± 2.2 (control veraison stage, VC clusters), and 26.2°C ± 2.5 (control ripe stage, RC clusters). HT increased the average daytime pulp temperature to 34.7 ± 1.4°C, 33.7 ± 1.9°C, and 35.2 ± 2.5°C for GHT, VHT, and RHT clusters respectively (Figure 1B). Depending on the developmental stage, this experimental set-up led to average pulp temperature differences of 8.2°C ± 1.3 (GHT vs. GC), 7.9°C ± 1.1 (VHT vs. VC) and 9.0°C ± 0.8 (RHT vs. RC) between heat-treated and control berries (Figure 1B).

**Figure 2:**
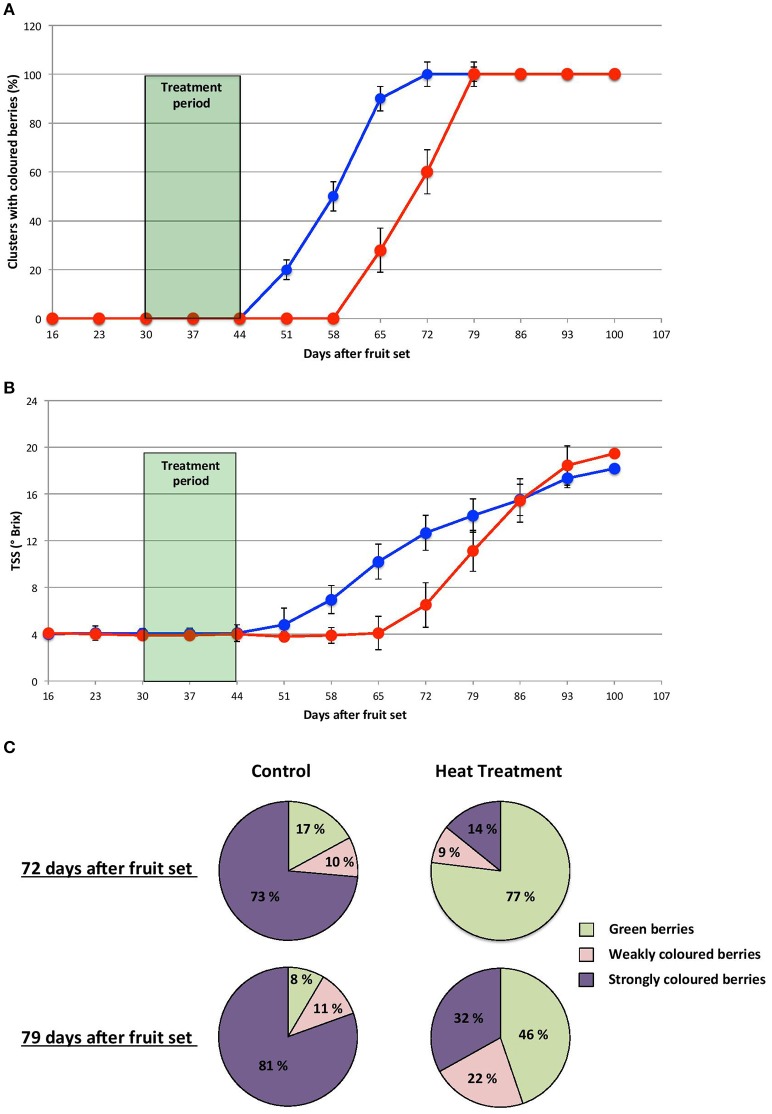
**Heat treatment imposed during the herbaceous stage delays the onset of veraison**. Proportion of colored clusters **(A)** and total soluble solids content **(B)** in developing control (blue line) and HT clusters (red line). **(C)** Proportion of turned berries in control and HT clusters, 72 and 79 days post fruit set. *n* = 25 clusters per condition.

The HT imposed during the herbaceous stage delayed the onset of anthocyanin accumulation by 2 to 3 weeks (GHT *vs* GC berries), (Figure 2A). Furthermore, for control clusters 4 weeks were needed to reach complete color turning stage (100% clusters (*n* = 25) while at least one additional week was required to reach the same stage in the heated clusters. However, even at the last stage, the percentage of uncolored berries per cluster (46%) remained much higher than in control clusters (8%) (Figure 2C). A similar delay in the increase in total soluble solids (TSS) content was also observed in stressed berries (Figure 2B) while the average berry weight was not affected by the treatments (data not shown).

### Biochemical analysis of the treated berries

The biochemical content of berries collected at harvest from control fruiting cuttings was compared with that of berries exposed to a 2-week period HT at 3 different developmental stages (Table 1). The berry hexose content (glucose and fructose) at harvest was not affected whenever the fruit-localized HT took place. By contrast, applying HT at veraison and to a lesser extent at ripening stage decreased malate content while tartrate content was slightly higher in VHT clusters than in other samples. In the present study, 20 amino acids including the non-proteinogenic ones were quantified. The concentration of 7 amino acids (THR, ARG, TYR, PHE, CYS, LYS, GABA) was significantly increased by a HT applied at veraison or ripening stages. Conversely, the PRO amount decreased dramatically after mid-ripening treatment (Table 1).

**Table 1:** **Effects of heat treatment on the contents of some relevant components of Cabernet Sauvignon berries at harvest**.

**Compounds**	**CT**	**GHT**	**VHT**	**RHT**
**Hexoses (g. L^−1^)**				
Glucose	118.3 ± 15.2^a^	117.6 ± 16.2^a^	127.3 ± 6.5^a^	106.3 ± 5.4^a^
Fructose	120.7 ± 14.6^a^	120.4 ± 15.8^a^	132.5 ± 7.4^a^	106.2 ± 5.9^a^
**Organic acids (g. L^−1^)**				
Malic acid	3.63 ± 0.41^a^	3.84 ± 0.54^a^	1.99 ± 0.13^c^	2.52 ± 0.27^b^
Tartaric acid	3.08 ± 0.15^b^	3.38 ± 0.15^b^	4.21 ± 0.02^a^	3.23 ± 0.14^b^
**Amino acids (μmoles. L^−1^)**				
Aspartic acid	323.5 ± 41.3^b^	384.0 ± 11.2^b^	486.7 ± 18.8^a^	361.7 ± 24.5^b^
Glutamic acid	979.8 ± 115.9^ab^	1077.9 ± 90.8^ab^	1104.9 ± 30.2^a^	890.3 ± 67.9^b^
Serine	22.4 ± 5.3^a^	22.9 ± 2.1^a^	25.9 ± 6.7^a^	24.0 ± 4.8^a^
Asparagine	330.3 ± 68.4^a^	345.8 ± 40.5^a^	357.8 ± 23.8^a^	426.9 ± 55.9^a^
Glycine	71.1 ± 36.7^a^	58.8 ± 16.9^a^	57.8 ± 5.8^a^	64.4 ± 5.4^a^
Glutamine	382.9 ± 190.0^a^	478.8 ± 95.7^a^	336.2 ± 116.8^a^	604.6 ± 103.2^a^
Histidine	136.5 ± 61.8^a^	188.2 ± 32.6^a^	179.3 ± 34.4^a^	224.0 ± 8.2^a^
Threonine	695.4 ± 242.7^c^	850.9 ± 389.4^bc^	3081.7 ± 128.8^a^	1254.6 ± 182.6^b^
Alanine	958.7 ± 294.0^a^	1100.2 ± 231.2^a^	1052.5 ± 75.8^a^	1119.4 ± 123.1^a^
Arginine	371.9 ± 49.7^a^	376.8 ± 60.6^a^	602.1 ± 68.3^b^	503.6 ± 38.7^b^
g-aminobutyric acid	907.9 ± 73.6^b^	1154.3 ± 356.7^b^	1594.1 ± 589.1^b^	4762.9 ± 2086.7^a^
Proline	25667 ± 2814^a^	22194 ± 1035^a^	27707 ± 4085^a^	9816 ± 2627^b^
Tyrosine	39.1 ± 7.1^b^	36.1 ± 7.2^b^	80.8 ± 25^ab^	136.7 ± 56.9^a^
Phenylalanine	41.9 ± 9.6^b^	37.8 ± 3.6^b^	55.5 ± 12.8^b^	155.5 ± 41.2^a^
Cysteine	19.4 ± 6.4^bc^	13.9 ± 12.0^c^	38.4 ± 9.7^ab^	50.8 ± 7.6^a^
Valine	271.7 ± 31.9^a^	321.2 ± 51.8^a^	357.1 ± 59.3^a^	350.5 ± 36.0^a^
Methionine	11.2 ± 6.7^a^	19.8 ± 4.0^a^	27.4 ± 9.8^a^	24, 8 ± 6.8^a^
Isoleucine	135.9 ± 12.7^a^	119.3 ± 15.4^a^	71.3 ± 11.6^b^	146.2 ± 20.8^a^
Leucine	180.4 ± 20.0^a^	163.3 ± 15.7^a^	159.9 ± 19.9^a^	234.9 ± 15.8^b^
Lysine	28.1 ± 5.9^b^	33.9 ± 6.3^b^	227.7 ± 40.6^a^	210.4 ± 28.3^a^
**Skin Anthocyanins (mg. g^−1^ skin DW)**				
Total skin anthocyanins (TSA)	19.41 ± 2.99^ab^	21.00 ± 6.52^a^	12.23 ± 2.10^ab^	10.92 ± 1.70^b^
Di-hydroxylated anthocyanins	4.70 ± 1.03^a^	2.34 ± 0.55^b^	1.04 ± 0.29^b^	0.96 ± 0.24^b^
Tri-hydroxylated anthocyanins	14.71 ± 1.89^a^	18.66 ± 2.91^a^	11.18 ± 1.99^a^	9.96 ± 1.70^a^
Ratio Di-/Tri-hydroxylated anthocyanins	0.32 ± 0.03^a^	0.13 ± 0.01^b^	0.09 ± 0.01^b^	0.10 ± 0.02^b^
Acylated anthocyanins (%)	24.5 ± 1.1^c^	33.8 ± 1.8^b^	40.4 ± 1.4^a^	37.5 ± 2.7^ab^
Malvidin-3-*O*-glucoside	5.74 ± 0.76^a^	7.77 ± 2.87^a^	4.52 ± 0.94^a^	4.34 ± 0.79^a^
Delphinidin-3-*O*-glucoside	2.12 ± 0.44^a^	1.17 ± 0.36^b^	0.21 ± 0.05^c^	0.37 ± 0.14^c^
Cyanidin-3-*O*-glucoside	1.23 ± 0.33^a^	0.33 ± 0.04^b^	0.04 ± 0.01^b^	0.07 ± 0.05^b^
Petunidin-3-*O*-glucoside	1.27 ± 0.23^a^	0.88 ± 0.37^ab^	0.25 ± 0.05^bc^	0.37 ± 0.09^c^
Peonidin-3-*O*-glucoside	2.62 ± 0.45^a^	1.42 ± 0.48^b^	0.70 ± 0.14^b^	0.71 ± 0.14^b^
Malvidin-3-*O*-(6′-acetyl) glucoside	3.34 ± 0.33^b^	6.04 ± 1.56^a^	4.61 ± 0.67^ab^	3.83 ± 0.81^ab^
Delphinidin-3-*O*-(6′-acetyl) glucoside	0.54 ± 0.07^a^	0.39 ± 0.14^a^	0.09 ± 0.02^b^	0.11 ± 0.02^b^
Cyanidin-3-*O*-(6′-acetyl) glucoside	0.33 ± 0.08^a^	0.16 ± 0.03^b^	0.05 ± 0.04^b^	0.06 ± 0.01^b^
Petunidin-3-*O*-(6′-acetyl) glucoside	0.54 ± 0.10^a^	0.44 ± 0.15^ab^	0.17 ± 0.03^bc^	0.23 ± 0.02^c^
Peonidin-3-*O*-(6′-acetyl) glucoside	1.15 ± 0.22^a^	0.99 ± 0.15^a^	0.81 ± 0.03^b^	0.24 ± 0.05^c^
Malvidin-3-*O*-(6′-*p*-coumaroyl) glucoside	1.09 ± 0.14^a^	1.88 ± 0.50^a^	1.28 ± 0.22^a^	1.23 ± 0.40^a^
Delphinidin-3-*O*-(6′-*p*-coumaroyl) glucoside	nd	nd	nd	nd
Cyanidin-3-*O*-(6′-*p*-coumaroyl) glucoside	0.09 ± 0.04^a^	0.04 ± 0.03^a^	0.03 ± 0.01^a^	0.04 ± 0.03^a^
Petunidin-3-*O*-(6′-*p*-coumaroyl) glucoside	0.08 ± 0.01^ab^	0.09 ± 0.02^a^	0.04 ± 0.01^ab^	0.07 ± 0.02^b^
Peonidin-3-*O*-(6′-*p*-coumaroyl) glucoside	0.44 ± 0.08^a^	0.38 ± 011^ab^	0.23 ± 0.03^b^	0.22 ± 0.05^b^

*Metabolite concentrations were determined around harvest. Means ± SE are shown (n = 3). Different letters indicate significant differences between control and stress conditions within a given compound at P < 0.05 accoring to a Tukey's test. CT, control; GHT, heat treatment applied during the herbaceous stage; VHT, heat treatment applied during veraison stage; RHT, heat treatment applied during the ripening stage. nd, not detected*.

The total anthocyanin concentration (TSA) in berry skin at harvest was reduced by about 50% in VHT and RHT when compared to control and GHT berries (Table 1). Interestingly, the GHT berries that colored later (Figure 1, Supplementary data) displayed a TSA comparable to control berries at harvest. Cabernet Sauvignon berries contained higher amounts of tri-hydroxylated anthocyanins than di-hydroxylated ones in both control and heated conditions. Heat exposure preferentially decreased the proportion of dihydroxylated anthocyanins, regardless of the period of HT treatment (Table 1). Acylated anthocyanins represented a higher proportion of the total anthocyanin pool in HT berries (Table 1). The concentration of malvidin-3-*O*-glucoside, which is the most abundant anthocyanin in Cabernet Sauvignon berries (Dimitrovska et al., 2011; Lorrain et al., 2011) remained unaffected at harvest whereas the amount of one of its acylated forms (malvidin-3-*O*-(6′-acetyl)-glucoside) was significantly increased in GHT clusters. By contrast, the concentrations of dephinidin, cyanidin, petunidin, peonidin and of their 3-acetyl-glucoside derivatives were severely reduced by heat irrespectively of the period of treatment in GHT, VHT and RHT berries. The inhibiting effect of HT on the corresponding 3-coumaroyl-glucoside derivatives was less pronounced.

### Alteration of global berry transcriptome in response to localized heat treatment

**Figure 3:**
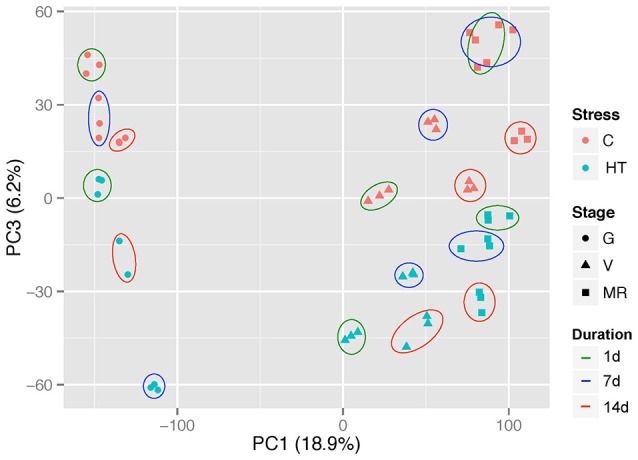
**Principal component analysis of the whole normalized gene expression dataset**. Red and blue symbols represent control and heated samples, respectively. Solid lines encircle the three replicates of each stage subjected to the same treatment, and the different color lines represent the treatment duration (1, 7, or 14 d). G, green; V, veraison; MR, middle ripening

Principal component analysis of the whole normalized gene expression dataset showed that the 3 replicates of each experimental condition are well-grouped and therefore adequate for further analysis (Figure 3; Supplementary Figure 1). Principal component 1 (PC1) and PC2 explained 26% of the total variance in gene expression and can be attributed to development. PC1 (18.9%) clearly separated the green stage from both veraison and ripening stages, whereas separation between veraison and the two other stages can be distinguished on PC2 (7.1%) (Supplementary Figure 1). PC3 explained 6.2% of the total variance and splits HT from control samples in a similar proportion whatever the developmental stage. Finally, the samples were clearly separated according to the treatment duration, potentially reflecting a developmental dependent response (Figure 3).

**Figure 4:**
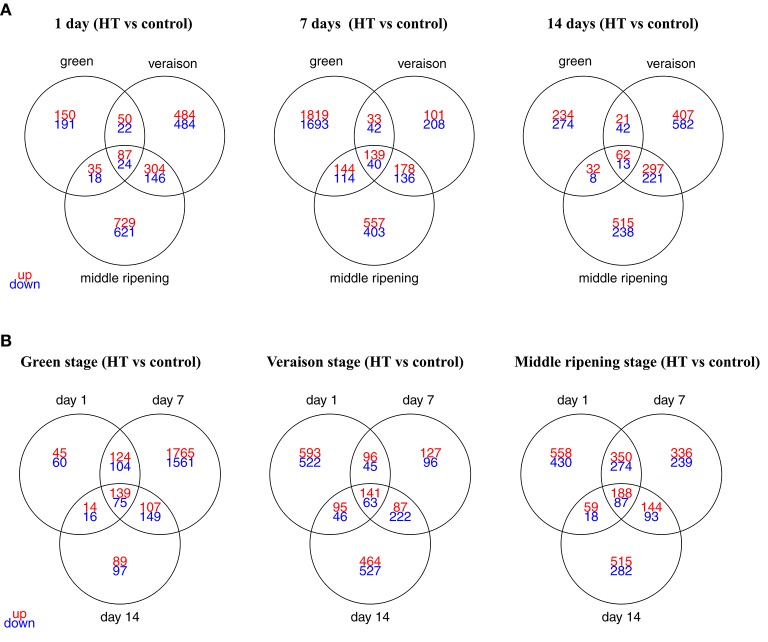
**Venn diagrams displaying the numbers of DEGs in heat-treated berries, according to the developmental stage (A)** or the treatment duration **(B)**. DEGs were selected using a 2-fold expression change and an adjusted *p* < 0.05 (with FDR correction). The detailed information of the common DEGs upon HT for each stage or for each exposure time was listed as in Supplementary Table 1.

A total of 7518 transcripts were differentially expressed (fold change > 2, *p*-value adj. < 0.05) in at least one of the 9 conditions (Supplementary Table 1), corresponding to 25.4% of the unigenes represented on the microarray slide. The overlaps in DEGs under these nine conditions were depicted with 3-way Venn diagrams, according to developmental stage (Figure 4A) or stress duration (Figure 4B). The strongest HT effect was observed for the herbaceous stage with 5287 DEGs, compared to 4122 and 5061 DEGs identified after HT exposure at veraison and ripening stage, respectively (Figure 4A; Supplementary Table 2). A total number of 4141, 6612, and 3717 genes were deregulated after 1, 7, and 14 days of HT, respectively (Figure 4B; Supplementary Table 2). Except for one condition (V14D), the number of up-regulated genes was always higher than the number of down-regulated genes. The strongest HT effect was observed at G7D with 4024 DEGs (2135 up-regulated and 1889 down-regulated genes, 7 and 6% of the grapevine unigenes, respectively) whereas the smallest effect (577 DEGs) was observed in G1D. The comparison of all 9 conditions showed that only 36 genes were steadily induced under HT condition whereas continuously down-regulated transcripts were not found (Supplementary Table 3). These genes will be further considered in the discussion section below.

### Identification of significantly altered functional categories according to the stage and the treatment duration

**Figure 5:**
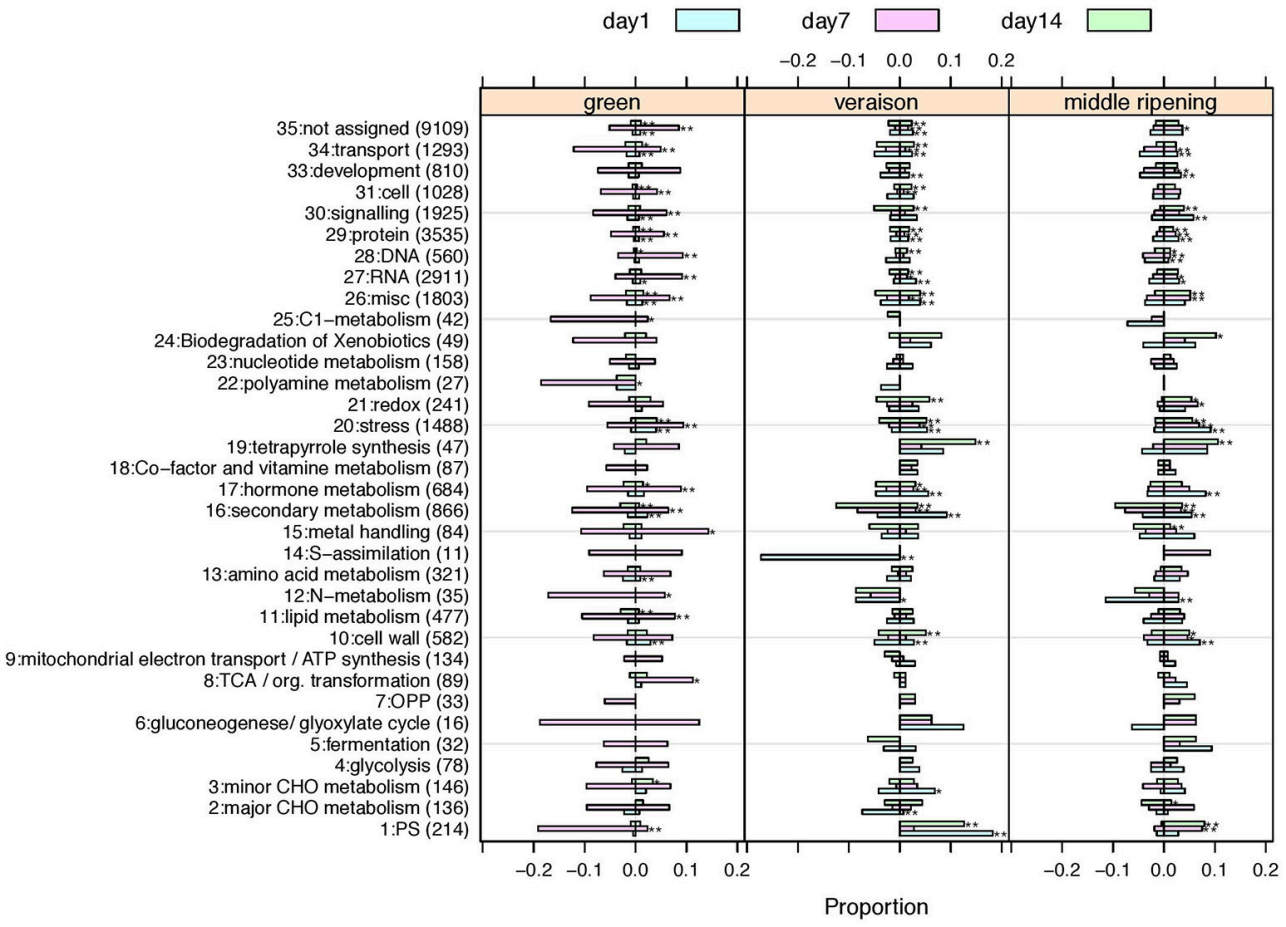
**Proportions of up- or down-regulated genes for each MapMan main category**. One panel per developmental stage is presented. Treatment durations are colored. Overproportionally affected categories based on a Chi-square test are marked by asterisks (^*^p < 0.01; ^**^p < 0.001.). Category number (BIN), description and the total number of associated genes are given on the y-axis. Negative values on the x-axis indicate the proportion of down-regulated genes, positive values the proportion of up-regulated genes. A tabular view also indicating the number of significantly impacted conditions per category and the number of up- or down-regulated genes is given in Supplementary Table 5.

To gain insight into the functional categories impacted by HT, the 7518 DEGs were distributed into 35 MapMan functional categories, the so-called BINs (Usadel et al., 2005). The proportion of DEGs representing each category was determined for each of the nine experimental conditions (Figure 5). Functional enrichment analysis was performed to better identify the significantly altered functional categories according to the stage and the stress duration. HT triggered a wide range of effects on the berry transcriptome (Figure 5; Supplementary Table 4). Twenty-seven of the 35 MapMan BINs were significantly altered in HT berries in at least one condition, but only 3 functional categories, namely “Stress” (BIN 20), “Protein” (BIN 29) and “Secondary metabolism” (BIN 16), were profoundly affected in all 9 conditions. The 24 other categories were differentially affected according to the developmental stage and the stress duration, and particularly correspond to Photosynthesis (BIN 1), Cell wall (BIN 10), Hormone metabolism (BIN 17), RNA (BIN 27), DNA (BIN 28), Signaling (BIN 30), and Transport (BIN 34).

#### “Stress” -associated BIN

Four hundred twenty seven DEGs were related to the functional category “Stress” and mainly belonged to the “abiotic/heat stress” cluster (Supplementary Table 5; 98 out of the 427 “Stress” DEGs). Most of the transcripts associated to “heat stress” category were predominantly up-regulated (Supplementary Figure 2A) and belong to the HSP family (Heat Shock Protein). Transcripts encoding universal stress proteins (USPs; VIT_17s0000g04260, VIT_04s0079g00610, VIT_08s0032g00590) also accumulated in response to HT.

#### “Protein” -associated BIN

HT deeply affects protein homeostasis. Indeed, numerous HSP and chaperones genes were up-regulated in HT berries (Supplementary Tables 5, 6). These proteins play an important role in protein-protein interactions (Kotak et al., 2007a; Bokszczanin and Fragkostefanakis, 2013). The abundance of several FK506-binding protein (FKBP) related transcripts was also increased by HT (VIT_19s0015g01100, VIT_07s0031g01150, VIT_08s0007g04340, VIT_13s0064g00580, VIT_01s0011g00930, VIT_00s0260g00070). These proteins belong to the large family of peptidyl prolyl cis–trans isomerases that can function as chaperones. Protein synthesis and degradation were also strongly affected in heated berries as suggested by the 60 DEGs linked to protein synthesis category and the 348 genes belonging to proteolysis (Supplementary Tables 5, 6).

Protein degradation through the ubiquitin-proteasome system (UPS) plays an essential role in diverse cellular pathways, cell-cycle progression, DNA repair, and degradation of damaged proteins as well as in signal transduction. Particularly, UPS components are major players in plant acclimation to abiotic stresses (Stone, 2014; Guerra et al., 2015). In the present study, 167 transcripts potentially linked to the ubiquitin machinery were deregulated after HT (102 up-, 56 down-, 9 both up- and down-regulated transcripts; Supplementary Table 6). Most of these DEGs encode different putative ubiquitin ligases (E3). Orthologs of each of the 3 major E3 classes (namely RING-type (Really Interesting New Gene); HECT-type (Homology to E6-Associated Carboxyl- Terminus), and U-box-type) were up-regulated in heat-stressed berries. While some E3-like transcripts accumulated whatever the developmental stage (VIT_09s0002g00220, VIT_12s0034g01390, VIT_05s0124g00230, VIT_06s0009g03670, VIT_08s0040g02600, VIT_19s0027g00320), most of these were transiently or specifically deregulated at a particular stage. For instance, some E3-related transcripts were only HT up-regulated during the herbaceous stage (VIT_17s0000g09790, VIT_18s0041g01090, VIT_14s0068g02150, VIT_14s0066g02580, VIT_08s0056g01410) whereas others responded to HT during veraison or ripening stages (VIT_19s0015g00660, VIT_01s0011g02950, VIT_01s0026g00300, VIT_07s0005g01360, VIT_08s0007g04790, VIT_18s0001g02280, VIT_00s0160g00270, VIT_18s0001g06220). Furthermore, the CRL group (Cullin based Ring E3 ligases), corresponding to the largest class of ubiquitin ligases (Stone, 2014), and especially the CUL1 based E3s, [also referred to as Skp1-Cullin-F-box (SCF)] were strongly impacted by HT. Indeed, an ortholog of the Arabidopsis adaptor protein S-Phase kinase-associated protein (SKP; VIT_03s0038g02480) and many F-box proteins are up-regulated at the transcriptional level upon HT.

#### “Secondary metabolism” -associated BIN

The secondary metabolism produces compounds of critical importance for berry quality and wine bitterness and astringency (Lund and Bohlmann, 2006; Ali et al., 2010; Kuhn et al., 2014; Robinson et al., 2014). Three hundred twenty seven DEGs were related to “Secondary metabolism” and mainly belonged to the subcategories “isoprenoids,” “phenylpropanoid-lignin,” and “flavonoids” (Supplementary Table 7).

Numerous genes affected by HT correspond to aroma and aroma-precursor related gene. Terpenes (predominantly eucalyptol, β-caryophyllene, and α-humulene) are usually present at low levels in Cabernet Sauvignon grapes, accumulating during the preveraison stage whereas benzene derivatives (2-phenylethanol and 2-phenylethanal) appear at late ripening (Kalua and Boss, 2009; Robinson et al., 2014). The strong repression of genes encoding the *1-deoxy-D-xylulose-5-phosphate synthase* (VIT_05s0020g02130, VIT_09s0002g02050, VIT_11s0052g01730, VIT_11s0052g01780) suggests that volatile terpenoids biosynthesis may be decreased by HT (Supplementary Table 7). The 1-deoxy-D-xylulose-5-phosphate synthase catalyzes the synthesis of isopentenylpyrophosphate (IPP), which is the precursor of all terpenes. The condensation of IPP and its isomer DMAPP (dimethylallylpyrophosphate) forms geranyl diphosphate (GPP) that is used by terpene synthase (TPS) to produce monoterpenes and derivatives. The Pinot Noir reference genome contains 89 putative *TPS*, among which half have been functionally characterized (Martin et al., 2010). Among the 55 probe sets giving reliable results and representing transcripts of functional, partial and pseudo *TPS* on the NimbleGen array (Cramer et al., 2014), 16 transcripts showed differential abundance in HT berries (Supplementary Table 8). Thirteen *TPS* were transiently repressed in GHT, VHT and or RHT clusters whereas only three *TPS* were upregulated by HT (*VviTPS25, VviTPS26, VviTPS50*). From the literature, most of these *TPS* accumulate at the late stages of ripening in vineyard conditions (Cramer et al., 2014). Finally, the transcript abundance of several terpene-related genes decreased in berries directly exposed to HT, in a similar way to that observed after exposing the whole vine to HT (Rienth et al., 2014). These repressed genes encode geraniol 10-hydroxylase, (-)-germacrene D synthase and linalool synthase (Supplementary Table 8).

Within the terpene family, carotenoids are a complex subgroup of isoprenoid pigments playing diverse roles in plants and providing nutritional value. Carotenoids also lead to C_13_-norisoprenoids which contribute the characteristic aromas of *Vitis vinifera* varieties (Mendes-Pinto, 2009). Among the 42 putative grape carotenoid metabolic genes (Young et al., 2012), 21 transcripts showed differential abundance after HT in at least one of the 9 conditions, most of these being down-regulated (Supplementary Tables 7, 9). The abundance of 2 out of 3 phytoene synthase transcripts (*VviPSY, VIT_12s0028g00960, VIT_06s0004g00820*), encoding the enzyme that catalyzes the first step committed to carotenoid biosynthesis, decreased upon HT. This down-regulation was also observed for downstream genes, namely phytoene dehydrogenase (VIT_04s0023g01790), carotene desaturase (VIT_14s0030g01740), carotenoid isomerase (VIT_08s0032g00800, VIT_12s0035g01080), lycopene cyclase (VIT_06s0080g00810, VIT_11s0016g01880) and carotene hydroxylase (VIT_04s0023g00080). The β-carotene hydroxylase 2 transcripts (*VviBCH2*, VIT_16s0050g01090) were the only ones strongly accumulating under HT, and especially at the herbaceous stage.

Methoxypyrazines (MPs) are strongly odorant volatile molecules with vegetable-like fragrances that participate to the distinct herbaceous/ bell pepper characters of some wines such as Cabernet Sauvignon (Dunlevy et al., 2009; Darriet et al., 2012; Kuhn et al., 2014). Isobutyl methoxypyrazine (IBMP) is the predominant MP in Cabernet Sauvignon berries, accumulating throughout the pre-veraison stage before declining during the ripening phase. The last step of its biosynthesis was recently deciphered in grapevine, through the unambiguous identification of VviOMT3 (VIT_03s0038g03090), an *O*-methyltransferase capable of converting the nonvolatile precursor 2-hydroxy-3-isobutylpyrazine (IBHP) into IBMP (Dunlevy et al., 2013; Guillaumie et al., 2013). By contrast to VviOMT4, VviOMT1 and VviOMT2 are active with a broad range of substrates but methylate IBHP *in vitro*, although with poor affinity (Dunlevy et al., 2010). Interestingly, *VviOMT3* transcripts were strongly repressed in green berries exposed to HT (Supplementary Table 7). A similar decrease was also observed in GHT berries for *VviOMT4* (VIT_03s0038g03080) and *VviOMT1* (VIT_12s0059g01790) transcripts, whereas *VviOMT2* (VIT_12s0059g01750) remained unaffected.

**Figure 6:**
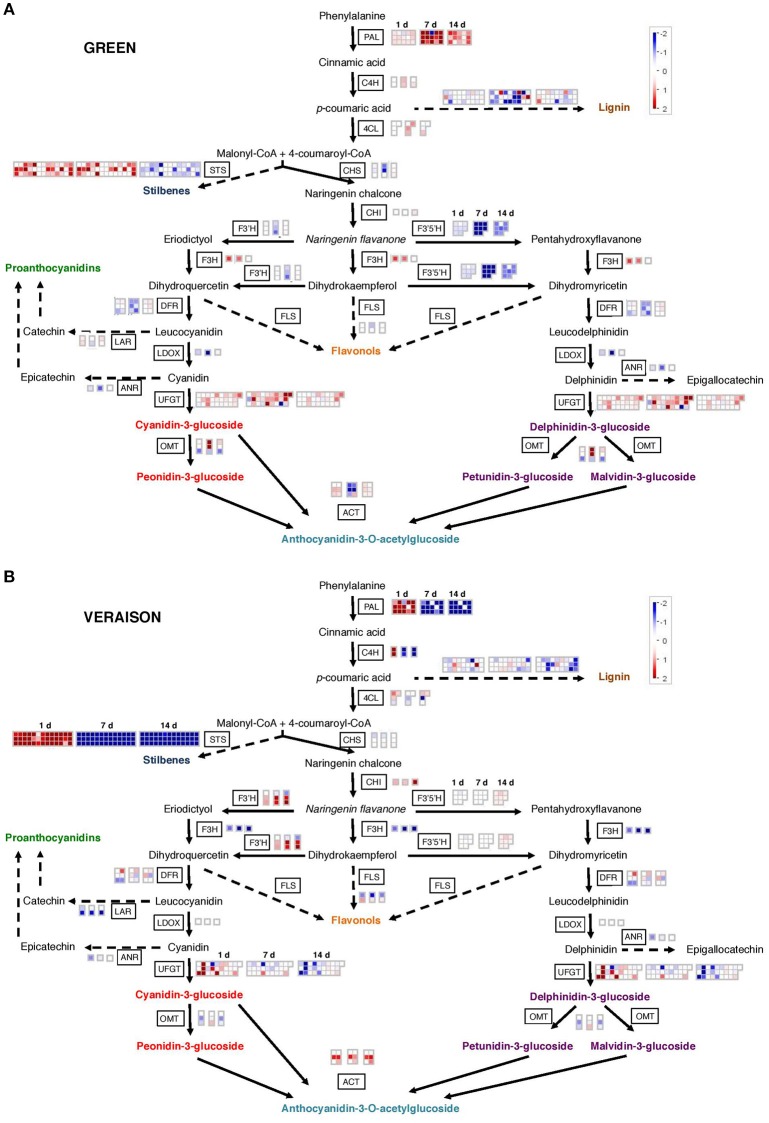
**MapMan visualization of heat differentially expressed genes associated to the phenylpropanoid pathway. (A)** The treatment was applied on green berries. **(B)** The treatment was applied at veraison. Each box corresponds to a treatment duration (1, 7, and 14 days). The expression ratio of each gene (log2 HT/Control) is represented by a colored square following the color scale. Red, expression up-regulated under HT; blue, expression down-regulated under HT; white, no significant differential expression. The effect of HT on the phenylpropanoid pathway in ripening clusters is given in Supplementary Figure 3.

Grape berry phenolics derived from the phenylpropanoid pathway participate to sensory properties, color and protection against environmental stress (Teixeira et al., 2013). Our biochemical analysis highlighted the dramatic effects of local warming on the onset of veraison and on the final anthocyanin contents in berries at harvest (Table 1; Figure 2). MapMan analysis (Figure 6) shows the contrasted heat responses of DEGs related to the general phenylpropanoid and to the flavonoid biosynthesis pathways (Figure 6; Supplementary Figure 3; Supplementary Table 10). The transcriptomic responses of GHT samples clearly differed from both VHT and RHT samples. During the herbaceous stage, genes involved in the general phenylpropanoid pathway (*phenylalanine ammonia-lyase PAL, cinnamate 4-hydroxylase C4H, 4-coumarate-CoA ligase 4CL*) were induced by HT regardless to the stress duration. By contrast, in VHT and RHT samples, the same genes were up-regulated after 1 day, but strongly repressed after 7 and 14 days of treatment. Many transcripts related to the flavonoid biosynthesis pathway were repressed in GHT clusters. Two *chalcone synthase* transcripts (*VviCHS1*: VIT_14s0068g00920, *VviCHS2*: VIT_14s0068g00930) encoding the first committed enzyme in flavonoid biosynthesis (Parage et al., 2012) were repressed after 7 days. A significant repression by HT was also observed for many flavonoid transcripts of the late biosynthetic pathway, such as *flavonoid 3*′*-hydroxylase* (*VviF3*′*H*, VIT_11s0016g01020, VIT_11s0016g01030, VIT_09s0002g01090*), flavonoid 3*′*5*′*-hydroxylase* (*VviF3*′*5*′*H*), *dihydroflavonol 4-reductase* (*VviDFR*, VIT_16s0039g02350, VIT_18s0001g1280) and *leucoanthocyanidin dioxygenase* (*VviLDOX*, VIT_08s0105g00380), whereas the *flavanone 3-hydroxylase* gene (*VviF3H*, VIT_16s0098g00860) was up-regulated (Figure 6; Supplementary Figure 3; Supplementary Table 10). The HT led to contrasted effects on the same set of genes in VHT and RHT clusters when compared with GHT. *VviF3H* transcript was significantly less abundant whereas *VviCHI* (chalcone isomerase, VIT_19s0014g00100) and *VviF3*′*H* genes were mostly up-regulated. Moreover, the repressive effect of HT on *VviF3*′*5*′*H* isoforms was not observed in VHT clusters and only transiently detected in RHT berries. In addition, the structural genes *VviANR* (*anthocyanidin reductase*, VIT_00s0361g00040), and *VviLAR* (*leucoanthocyanidin reductase*, VIT_01s0011g02960, VIT_17s0000g04150) involved in proanthocyanidins (PA) synthesis were significantly repressed under HT, according to the stage and/or duration of the stress.

The anthocyanidin aglycones are further modified through glycosylation, methylation and acylation events, leading to the production of a wide variety of anthocyanin compounds. Glycosylation of flavonoids is catalyzed by enzymes from the large glycosyltranferase (GT) family, represented by 240 genes in the grape genome (Ono et al., 2010). Glycosylation enhances the structural diversity and modifies the functional properties of these secondary metabolites. For instance, the expression of *UFGT* [*UDP-glucose:flavonoid 3-O-glycosyltransferase*, renamed *VviGT1*, VIT_16s0039g02230 (Parage et al., 2012)], catalyzing the 3-O-specific glycosylation of anthocyanidin is critical for the coloration of grape skin (Boss et al., 1996). Twenty-nine putative *VviGT* were deregulated in berries exposed to HT (Figure 6; Supplementary Figure 3; Supplementary Table 10). Despite the loss of anthocyanins upon HT, *VviGT1* expression remained unaffected by HT. By contrast, *VviGT5* (VIT_11s0052g01600) and *VviGT6* (VIT_04s0023g01290) transcripts were strongly down-regulated in VHT and RHT clusters. *VviGT5* and *VviGT6* drive the glycosylation of flavonols, which increase their water solubility and their accumulation (Ono et al., 2010).

In grapevine, anthocyanins can also be modified through the action of *O*-methyltransferases (AOMTs) and acyltransferases (ACTs) before being transported into the vacuole (Fournier-Level et al., 2011; Rinaldo et al., 2015). These modifications modulate berry color by reducing anthocyanin reactivity and increasing their stability and solubility in water. None of the two grape *AOMTs* (*VviAOMT1*; VIT_01s0010g03510, *VviAOMT2*; VIT_01s0010g03490) that were previously described as effective anthocyanin 3′- and 3′,5′-*O*-methyltransferase (Hugueney et al., 2009; Lücker et al., 2010; Fournier-Level et al., 2011) was significantly deregulated by HT (Supplementary Table 10). However, other putative *VviAOMTs* transcripts (VIT_11s0016g02610, VIT_07s0031g00350, VIT_03s0063g00140, VIT_12s0028g03110), were transiently up- or down-regulated by HT depending on the ripening stage and stress duration. HT also modified the transcript amounts of various *ACTs* (Supplementary Table 10). Indeed, 5 putative *ACTs* were transiently repressed in GHT berries (VIT_12s0134g00590, VIT_12s0134g00630, VIT_12s0134g00600, VIT_12s0134g00650, VIT_12s0134g0660), whereas 3 *ACTs* (VIT_03s0017g00870, VIT_12s0134g00590, VIT_12s0134g00630) were up-regulated in both VHT and RHT berries. VIT_03s0017g00870 corresponds to Vvi3AT, an enzyme recently associated to the production of the common grape berry acylated anthocyanins (Rinaldo et al., 2015).

Stilbenes and lignins represent branching points in the phenylpropanoid pathway. Stilbenes are a small family of phytoalexins synthesized by plants in response to biotic and abiotic stresses. Under normal growth conditions, berries stilbene content increases from veraison to ripening, with significant differences among *V. vinifera* varieties (Gatto et al., 2008). Forty-eight *stilbene synthases (STSs)* genes catalyzing the biosynthesis of the stilbene backbone were found in grapevine genome. This represents an unusual example of functional redundancy (Parage et al., 2012). Most of these (39 genes) were impacted in berries exposed to HT, displaying a quite similar and noticeable expression profile (Figure 6; Supplementary Figure 3; Supplementary Table 10). In GHT clusters, 18 *STS* transcripts accumulated after 1 and 7 d of treatment before being repressed at 14 d. In VHT and RHT fruits, the *STSs* were transiently induced at 1 d and then strongly repressed over the 2 weeks of experiment.

Lignification can also be induced as a response to various biotic and abiotic stresses, as shown in Citrus fruit after postharvest HT (Yun et al., 2013). Conversely, the cell wall lignin content was reduced in the skin of grape mature berries experiencing a water stress (Vannozzi et al., 2012; Fernandes et al., 2015). In the present study, numerous transcripts (39) potentially involved in the lignin biosynthetic pathway differentially accumulated in HT berries (Figure 6; Supplementary Figure 3; Supplementary Table 10). Two *cinnamoyl-CoA reductase* (*VviCCR*) gene isoforms were affected in an opposite way by HT, VIT_14s0066g01150 being induced, and VIT_09s0070g00240 being repressed. In GHT and VHT clusters, the transcript levels of 16 *cinnamyl alcohol dehydrogenase* (*VviCAD*) genes were mainly repressed whereas a few ones accumulated in RHT berries. The content of 3 *ferulate 5-hydroxylase* transcripts (*VviF5H*) was reduced in both VHT and RHT berries. Finally, HT led to contrasted effects on *caffeic acid O-methyltransferase* (*VviCOMT*) and peroxidase transcripts (catalyzing the polymerization of monolignols into lignins).

While many structural genes were transcriptionally affected in HT berries, the positive regulatory genes identified so far in grapevine were either weakly (*VviMYBPA1, VviMYB14, VviMYB15, VviMYBF1*) or not deregulated (*VviMYBA1, VviMYBA2, VviMYBA3, VviMYC1, VviMYCA1, VviMYBPA2*) in HT berries (Supplementary Table 11). Accordingly, HT did not affect the amount of *VviGT1* transcript (VIT_16s0039g02230) that encodes the anthocyanidin glycosyltransferase catalyzing the limiting step for anthocyanin accumulation and are a direct target of VviMybA1 transcription factor (TF; VIT_02s0033g00410) (Cutanda-Perez et al., 2009). For comparison, no effect of HT was observed on *VviMYBA1* transcripts in berries from fruiting cuttings (Carbonell-Bejerano et al., 2013), whereas a *VviMYBA1* repression was reported in two others studies (Yamane et al., 2006; Rienth et al., 2014). It is also noteworthy that both *VviMYBC2-L3* (VIT_14s0006g01620) and *VviMYB4b* (VIT_04s0023g0371) transcripts were transiently enhanced in VHT and RHT berries, respectively. These two TFs were recently described as negative regulators of the phenylpropanoid pathway in grape (Cavallini et al., 2015). While HT did not significantly impact the expression of genes known to act in the transport of flavonoids (reviewed by Zhao (2015), either directly (*VviAM1, VviAM3, VviABCC1, VviMATE1, VviMATE2)* or indirectly *(VviGST1, VviGST4)*, the present work pointed out a significant deregulation of many putative ABC or MATE transporters and Glutathion S-transferase family members (Supplementary Tables 1, 11). It cannot be excluded that these proteins could be involved in the control of flavonoid transport in HT grape berries, as well.

In addition to synthesis, stabilization and vacuolar sequestration, the anthocyanin content can also be modulated through degradation that may involve enzymes such as laccases, polyphenol oxidases, class III peroxidases, and β-glucosidases (Oren-Shamir, 2009). Our experiments showed a strong HT effect on the expression of laccase genes, potentially impacting the polymerization rate of various phenolic compounds. Out of the 93 *laccase*-annotated genes in the grapevine genome, 33 transcripts were deregulated by HT (Supplementary Table 12). Interestingly, the laccase *TT10* (*Transparent Testa 10*, VIT_18s0075g00600) showed a maintained up-regulation by HT whatever the stage of development. In Arabidopsis, *TT10* was proposed to participate in the oxidative polymerization of phenolic compounds (Pourcel et al., 2005). In a similar way, numerous genes encoding putative polyphenol oxidases (4), peroxidases (25) and β-glucosidases (16) were impacted by HT (Supplementary Table 12). In the present study, HT induced several *peroxidase* genes including class III type (VIT_07s0130g00220, VIT_18s0001g06850) whereas others were repressed (VIT_18s0001g06840, VIT_18s0001g06890). Recently, Movahed et al. (2016) showed that the expression of 3 of the 5 *peroxidase* genes (VIT_14s0066g01850, VIT_06s0004g07770, VIT_07s0191g00050, VIT_11s0016g05320 and VIT_18s0072g00160) that were most strongly expressed in grape berry pericarp during ripening was influenced by temperature elevation. In agreement with these results, the present data also pinpoints the alteration of 4 of these genes (VIT_14s0066g01850, VIT_06s0004g07770, VIT_11s0016g05320 and VIT_18s0072g00160) in response to HT but with different kinetics and intensities.

## Discussion

### Locally applied HT delays the onset of veraison and alters berry composition at maturity

The temperature differences used in this experiment (Figure 1) are within the range of values obtained in vineyards between sun-exposed and shaded berries. For instance, the temperature of exposed Merlot berry can exceed air temperature by more than 10°C above ambient temperature, especially for clusters directly exposed to solar radiation (Pieri and Fermaud, 2005).

The present work highlights the strong inhibiting effects of HT (~ 35°C) locally imposed to clusters, on fruit-specific processes required for the onset of veraison. Conversely, damping diurnal berry temperature fluctuations (daytime cooling and night-time heating of clusters starting from fruit set) advanced the onset of ripening (Cohen et al., 2008; Tarara et al., 2008), as well as separated treatments (daytime cooling or night-time heating) even though the effects were less marked in the latter case (Cohen et al., 2012).

High temperatures promote the decrease of organic acid content observed after veraison, by exacerbating the malic acid breakdown (Ford, 2012; Rienth et al., 2016). Elevated temperatures accelerate the utilization of malate, enhancing the anaplerotic capacity of the TCA cycle for amino acid biosynthesis (Sweetman et al., 2014). By contrast to previous reports describing an increase in berry concentrations of various amino acids after exposure of whole grapevines to HT, the present data were obtained after berry-localized treatments. After a 14 d HT of whole grape fruiting cuttings, Carbonell-Bejerano et al. (2013) observed in fruits an increase in the content of 5 amino acid including TYR and PHE. Similarly, Sweetman et al. (2014) reported a boost in the berry concentration of 10 amino acids, among which THR, PRO and GABA, after applying at veraison an 11 d HT to potted Shiraz vines. The accumulation of amino acids is a widespread phenomenon of higher plants response to various abiotic stresses, including HT (Krasensky and Jonak, 2012; Bokszczanin and Fragkostefanakis, 2013). This increase may result from an up-regulation of amino acid synthesis, a decreased amino acid catabolism, and/or of an enhanced of stress-induced protein breakdown or to a change in amino acids imported from the plant. In grape berries exposed to HT, Sweetman et al. (2014) suggested that the amino acid accumulation was rather due to *de-novo* biosynthesis than to proteolysis. Among others, the increased levels of PRO and GABA may have a beneficial effect upon exposure to environmental cues (Krasensky and Jonak, 2012; Bokszczanin and Fragkostefanakis, 2013). PRO, which might act as a compatible osmolyte, a free radical scavenger and a protein chaperone, has been reported to have a protective role against abiotic stresses in many plant species. By contrast, tobacco and Arabidopsis plants do not accumulate PRO under HT and an excess of PRO reduces thermotolerance in Arabidopsis (Lv et al., 2011). PRO hyper-accumulated in Shiraz berries submitted to warming (Sweetman et al., 2014), but not in heat- treated Muscat Hambourg berries (Carbonell-Bejerano et al., 2013). PRO content was even strongly reduced in Cabernet Sauvignon fruits as shown in the present study (Table 1). These differences may be due to different experimental procedures (local vs whole plant treatments) and/or result from variety-dependent responses. Therefore the exact role of PRO in grape berry adaptation to HT remains to be determined.

The amount of the osmolyte GABA also increased in RHT berries (Table 1). This compound may act as a signaling molecule and affect different processes, including the control of the carbon–nitrogen balance and the protection against oxidative stress (Fait et al., 2007; Krasensky and Jonak, 2012). Recently, Sweetman et al. (2014) showed that a GABA shunt was up-regulated in warmed grape berries. This shunt is important for stress tolerance when the TCA cycle is down-regulated (Fait et al., 2007).

PHE and TYR whose concentration also increased in VHT and RHT berries (Table 1) are synthesized via the shikimate pathway followed by the branched aromatic amino acid metabolic pathway. Beside their role as building blocks of proteins, PHE and TYR serve as precursors for a wide range of secondary metabolites. Particularly, PHE is the substrate of PAL, the key enzyme of phenolic biosynthesis. The accumulation of PHE in HT berries may result from the strong repression of *PAL* genes observed during ripening, and to a larger extent, to the repression of genes encoding enzymes involved in the general phenylpropanoid pathway (Figure 6; Supplementary Table 7). Dai et al. (2014) reported that the up-regulation of the phenylpropanoid pathway genes and the increased accumulation of anthocyanins in response to high sugar supply is paralleled by a decrease in berry PHE content.

Heat exposure reduced the concentration of total anthocyanins and particularly the amount of dihydroxylated anthocyanins, and increased the proportion of acylated anthocyanins (Table 1). This result agrees with previous studies showing that application of elevated temperatures (>30°C) to fruits results in anthocyanin degradation and inhibition of their accumulation. The intensity of this phenomenon depends on the type of anthocyanin derivative and on the grape variety (Kliewer and Torres, 1972; Spayd et al., 2002; Mori et al., 2007; Cohen et al., 2008; Tarara et al., 2008; Azuma et al., 2012).

Altogether, the experimental design set up with Cabernet Sauvignon fruiting cuttings to address the direct impact of elevated temperatures on berry development provided reliable biochemical results that are comparable to those observed in vineyards or using potted grapevines. Moreover, the alterations observed in heated berries directly result from the cluster exposure to HT. Finally, while veraison is delayed in GHT berries, their biochemical contents at harvest are comparable to that observed in control fruits.

### Locally applied HT deeply affects grape berry transcriptome and triggers adaptative responses

Our results indicate that a local HT application deeply affected the berry transcriptome whatever the stage and the stress duration (Figure 4). The amplitude of the response is consistent with previous studies reporting that about 5% of the plant transcriptome is up-regulated 2-fold or more in response to HT (Mittler et al., 2012). The berry transcriptome varied with a similar amplitude when microvines were subjected to a short 2 h stress period during the day, whereas gene expression changes were more pronounced when this 2 h-HT was applied during the night (Rienth et al., 2014). The present work also revealed that only few genes were heat-deregulated whatever the developmental stage and the stress duration. The comparison of all 9 conditions showed that only 36 genes were steadily induced under HT condition whereas continuously down-regulated transcripts were not found (Supplementary Table 3). Not surprisingly, 24 genes encoding various HSPs are listed among the permanently up-regulated genes. Other genes correspond to transcripts encoding putative transcription factors (TFs) from the MYB (VIT_08s0007g06180) and the AP2/ERF (VIT_15s0046g01390) families. These TFs contrast with many other TFs that are deregulated in at least one experimental condition (602 DEGS, Supplementary Table 13), and take part in the observed deep remodeling of the berry transcriptome under heat. Especially, the function of AP2/ERF members in plant abiotic stress responses is clearly established (Mizoi et al., 2012) and few recent works suggested a similar role for this TF family in grapevine (Carbonell-Bejerano et al., 2013; Zhu et al., 2013; Rocheta et al., 2014). Two others transcripts coding for a sterol O-acyltransferase (VIT_00s2300g00010) and a truncated receptor-like kinase (VIT_00s0437g00010) respectively, showed disrupted expression levels in all the nine conditions (Supplementary Table 3). The closest ortholog of VIT_00s2300g00010 in Arabidopsis corresponds to *AtASAT1* (*Acyl-CoA Sterol Acyltransferase1*), an enzyme catalyzing the phytosterol ester biosynthesis in seeds (Chen et al., 2007). The precise role of this enzyme in the context of heat-induced responses remains to be determined. Finally, *VviGOLS1* (VIT_07s0005g01970) encoding a galactinol synthase (GOLS) was consistently up-regulated by HT, confirming our previous work (Pillet et al., 2012).

#### Heat-induced adaptive responses in berries

Essential for cell growth and viability, HSPs function as molecular chaperones in maintaining protein quality and folding, and are required for the acquisition of the thermotolerance (Bokszczanin and Fragkostefanakis, 2013). A massive up-regulation of *HSP* genes is a highly conserved response in heat-exposed plants (Finka et al., 2011). This response is a result of the complex signaling cascade, whose final steps consist in the activation of heat shock TFs (HSFs) and their binding to the *HSP* promoters (Mittler et al., 2012). HSF activity is regulated at the transcriptional, post-transcriptional and post-translational levels (Mittler et al., 2012) and 21 Arabidopsis *HSF* transcripts are up-regulated in response to various environmental stress conditions (Miller and Mittler, 2006). Data mining of the 12X version of the grape genome allowed the identification of 19 putative *VviHSFs* (Pillet et al., 2012; Scharf et al., 2012) among which 6 were up-regulated in HT berries (Supplementary Table 14). Among these, VIT_04s0008g01110 corresponds to *VviHSFA2* that was recently reported as involved in the regulation of heat responses in stressed berries (Pillet et al., 2012). HSFA2 is a key player for basal and acquired thermotolerance, extending the effect of heat acclimation in both tomato and Arabidopsis (Charng et al., 2007). AtHSFA2 which is the most highly HT-induced HSF (Busch et al., 2005), serves as a regulatory amplifier of a subset of genes (Schramm et al., 2008) and is required for the induction and maintenance of HT memory-related genes (Lämke et al., 2016). The transcriptional regulation of the heat-induced responses may also be due to *VviMBF1c* (Multiprotein bridging factor 1c; VIT_11s0016g04080) that showed enhanced expression pattern in HT berries (Supplementary Table 14), in good agreement with two recent studies (Carbonell-Bejerano et al., 2013; Rienth et al., 2014). In Arabidopsis, AtMBF1c acts as a thermotolerance mediator through the heat-dependent regulation of 36 different transcripts (Suzuki et al., 2011). The existence of a similar MBF1c regulon was proposed in ripening berries of microvines submitted to HT (Rienth et al., 2014). In the present study, the relationship between VviMBF1c and putative members of this regulon was not so tight, probably due to a much longer heat exposure (14 days *vs*. few hours).

Several transcripts encoding putative USPs also accumulated in response to HT (Supplementary Table 5). The USP family plays an important role in stress resistance in bacteria (Kvint et al., 2003). Based on expression profiles, a similar role in abiotic stress tolerance was suggested for various plant USPs (Kerk et al., 2003; Maqbool et al., 2009; Isokpehi et al., 2011). Recently, the Arabidopsis AtUSP was shown to exhibit a redox-dependant chaperone function and to enhance plant tolerance to heat shock and oxidative stress (Young Jun et al., 2015). The role of USPs in grapevine remains to be elucidated.

#### Protein homeostasis in heated berries

The maintenance of protein homeostasis, which includes the control of synthesis, intracellular sorting, folding, the function and degradation of proteins, is fundamental to ensure growth and development of plants under normal and stressful environmental conditions. In Arabidopsis, FKBP62 (ROF1) and FKBP65 (ROF2) are involved in acquired thermotolerance through the interaction with HSP90.1 and HSFA2 (Meiri and Breiman, 2009; Meiri et al., 2010). ROF1 contributes to the transcription activity of HSFA2 but ROF2, in the presence of ROF1, abolishes this activity. This suggests that ROF2 acts as a HT modulator through a negative feedback regulation of HSFA2 (Meiri et al., 2010). While expression of the grapevine *VviROF1* ortholog (VIT_00s0769g00010) was not affected in our experiments (Supplementary Table 6), *VviROF2* transcripts (VIT_00s0260g00070) accumulated upon HT at green and ripening stages (G7D, R1D and R7D). Half of the DEGs linked to the functional category “protein synthesis” encode ribosomal proteins known to prevent inhibition of protein synthesis under HT (Muñoz and Castellano, 2012). A massive heat-dependent deregulation of ribosomal protein genes was also described in wheat (Qin et al., 2008). Interestingly, one of the constantly and strongly up-regulated genes encodes a chloroplast ribosomal protein (RPS1; VIT_13s0101g00050), a heat responsive protein involved in retrograde activation of heat responses in Arabidopsis (Yu et al., 2012). Particularly, RPS1 was proposed as a critical factor in the activation of HSFA2 and of its target genes. Many genes related to protein targeting (34 DEGs) were also affected in HT berries and mainly up-regulated during the green stage (Supplementary Table 6).

HT-induced protein degradation is a complex process involving a multitude of proteolytic pathways, with many DEGs encoding cysteine protease, serine protease, arginine protease, subtilase or metalloprotease activities. For instance, 24 transcripts encoding AAA/FtsH metalloproteases were deregulated in heated berries (Supplementary Table 6). This protease family is essential for the protein quality control of mitochondrial and chloroplastic membranes, and to prevent damages caused by stress conditions (Janska et al., 2013). For example, *VviFtsH6* transcripts (VIT_14s0108g00590) strongly accumulated in berries when the HT was applied during the herbaceous stage. In Arabidopsis, the FtsH6 protease is involved in the degradation of the light-harvesting complex II (LHC II) during high light acclimatization and dark-induced senescence (Zelisko et al., 2005). The involvement of FtsH proteases in the quality control of the photosystem II was also described under moderate HT (Yoshioka et al., 2006) and a similar role may be attributed to FtsH6 in green photosynthetic berries exposed to HT. Another metalloprotease encoding gene (VIT_03s0088g00320) was highly up-regulated by HT at all stages. This transcript encodes an Arabidopsis *EGY3* ortholog (At1g17870) belonging to the EGY family (ethylene-dependent gravitropism-deficient and yellow-green-like 3). *AtEGY3* is an AtHSFA2 target gene (Nishizawa et al., 2006). The involvement of this putative membrane and plastidial metalloprotease in heat responses was recently pinpointed (Laranjeira and coworkers, unpublished, PhD thesis; http://hdl.handle.net/1822/18268). Both *FtsH6* and *EGY3* genes were also recently identified as heat responsive genes in *Brassica napus* siliques (Yu et al., 2014). The deregulation of numerous E3 ligase genes in HT berries may reflect an important role in triggering adaptive responses. Indeed, recent findings highlighted the role of various E3 ligases in mediating abiotic stress tolerance in Arabidopsis and in different crop species (Stone, 2014; Guerra et al., 2015). Most stress-related E3 ligases identified so far facilitate responses to environmental stimuli by modulating the abundance of key downstream stress-responsive TFs, thus affecting stress-related changes in gene expression. A non-proteolytic function of ubiquitin modification has also been reported in abiotic stress tolerance (Stone, 2014). For example, the rice E3 ligase OsHCI1 (Heat and Cold induced 1) monoubiquitinates some nuclear proteins in plants exposed to HT, which results in their translocation to the cytoplasm, and promotes HT tolerance (Lim et al., 2013). In apple, the E3 ligases MdCOP1s negatively regulate the peel anthocyanins content of fruits by modulating the degradation of the MdMYB1 protein (Li et al., 2012). A similar role can be envisaged in heat exposed berries to control the stability of flavonoid associated TFs. Many E2 conjugating enzyme genes are stress-inducible (Stone, 2014; Guerra et al., 2015). Two E2 transcripts were up-regulated when HT was applied on ripening berries. The first one (VIT_06s0004g08200) is an E2 enzyme ortholog of the Arabidopsis ubiquitin-conjugating enzyme 28 (UBC28, At1g64230). The second one (VIT_18s0001g10100) encodes a SUMO Conjugating Enzyme (SCE1a) binding small ubiquitin-like modifiers (SUMOs) to a wide range of cellular proteins. SUMOylation of AtHSFA2 represses its transcriptional activity and SUMO overexpression decreases sHSPs accumulation after HT (Cohen-Peer et al., 2010).

#### Heat treatment potentially decrease berry phenolic quality and aromatic potential

Low temperatures are beneficial to aroma production in the cool climate white grape cultivars (Duchêne and Schneider, 2005). Conversely, the aromatic potential of berries exposed to HT may be reduced (Belancic et al., 1997; Falcão et al., 2007), possibly due to heat-induced transcriptional changes (Rienth et al., 2014). Cabernet Sauvignon berries contain various aromatic molecules such as terpenes and C_13_-norisoprenoids (Robinson et al., 2014) and are typified by specific volatile thiols and methoxypyrazines (Bouchilloux et al., 1998). Our transcriptomic data indicate that direct exposure of Cabernet Sauvignon berries to HT may decrease their aromatic potential through deregulation of numerous aroma and aroma precursor-related genes (Supplementary Tables 7, 9). Indeed, volatile terpenoids biosynthesis may be decreased by HT, as suggested by the heat repression of many key enzymes of the biosynthetic pathway (1-deoxy-D-xylulose-5-phosphate synthase, terpene synthase, geraniol 10-hydroxylase, (-)-germacrene D synthase and linalool synthase). Comparable results were obtained after exposure of the whole vine to HT (Rienth et al., 2014). Our data also revealed that most of 21 DEGs linked to carotenoid metabolism were down-regulated after HT, with the exception of *VviBCH2*. In Arabidopsis, overexpression of *BCH2* improved tolerance to high light and HT by catalyzing the conversion of β-carotene to zeaxanthin and therefore preventing membranes from oxidative damage (Davison et al., 2002). While a constant decline in carotenoid abundance is generally observed after veraison in various cultivars including Cabernet Sauvignon (Deluc et al., 2009), our results suggest that HT may contribute to the decrease of carotenoid (except zeaxanthin) concentration before veraison, and/or accelerate its decrease after veraison. However, the possible consequences in term of aroma potential are not clear since only one type of CCD transcripts (*VviCCD4a*, VIT_02s0087g00910) was decreased by HT. Another gene (*VviCCD4b*, VIT_02s0087g00930) was transiently up-regulated by HT at veraison. The CCD enzymes convert their carotenoid substrates to C_13_-norisoprenoids, which encompass desirable flavor and aroma compounds in grapes and wine. In normal growth conditions, up-regulation of *VviCCD4a* and *VviCCD4b* was observed during ripening in Sauvignon Blanc and Pinotage (Young et al., 2012; Lashbrooke et al., 2013), whereas *VviCCD4a* expression pattern during berry development was dependent on the growth temperature regime applied to Cabernet Sauvignon fruit cuttings (Guillaumie et al., 2011). Carotenoid concentration in grape berries is influenced by microclimate through an effect of light exposure on the cluster (Kwasniewski et al., 2010; Young et al., 2015) and possibly through an impact of the temperature as suggested by our results. Finally, the present work highlights the HT repressive effects on 3 out of 4 *VviOMTs* (Supplementary Table 7), including VviOMT3, responsible for the synthesis of the predominant methoxypyrazine IBMP (Guillaumie et al., 2013). Natural (climate, soil) and viticultural factors impact on IBMP concentration in grapes and wines, and grapes ripened under HT produce wines with reduced IBMP contents (Darriet et al., 2012). Considering the strong correlation between *VviOMT3* expression and berry MP content, our data implies that HT lead to a strong reduction in IBMP synthesis during the herbaceous stage, resulting from the repression of the key gene *VviOMT3*, thus drastically reducing IBMP content in ripe berries.

Grape berry phenolic compounds contribute to organoleptic properties, color and protection against environmental cues. Our data show that a local warming of developing berries strongly affects the phenylpropanoid metabolic pathway. The effects depend on the stress duration and on the developmental stage (Figure 6; Supplementary Figures 2B, 3). Interestingly, the repression of *VviPAL* genes that correlated well with the accumulation of its substrate PHE and the deregulation of genes involved in anthocyanin stabilization (*VviAOMTs* and *VviACTs*) may contribute to the significant decrease of anthocyanin contents in HT berries at harvest (Table 1). Therefore, the delayed onset of veraison observed for GHT clusters could be considered at the transcriptional level. Additionally, together with the repression of *VviFLS1* (flavonol synthase, VIT_18s0001g03470; also named *FLS4*, Fujita et al., 2006; Czemmel et al., 2009), the down-regulation of *VviUFGT* genes (*VviGT5* and *VviGT6)* may contribute to the decrease of the flavonol content in heated berries. This result agrees with a recent work reporting the repression of *VviGT5* in detached berries subjected to elevated temperatures (Loyola et al., 2016). Since previous work reported little or no effect on skin flavonol amounts (Spayd et al., 2002), the consequences of a locally applied HT on flavonol content still have to be determined. The transcriptional regulation of many flavonoid structural genes under HT suggests a control through the associated TFs. Since no or weak effect was observed on the expression of the positive TFs identified so far (Supplementary Table 11), several hypothesis can be proposed among which an up-regulation of negative regulators such as *VviMYBC2-L3* and *VviMYB4b* (Supplementary Table 11; Cavallini et al., 2015), and a post-transcriptional control of the flavonoid associated genes as observed for the light-controlled stability of MYB1 protein in apple (Li et al., 2012).

A relationship between increased anthocyanin catabolism and elevated temperatures was proposed for grape berries (Mori et al., 2007). Although it is poorly known, anthocyanin degradation may involve enzymes such as laccases, polyphenol oxidases, class III peroxidases, and β-glucosidases (Oren-Shamir, 2009). In plants, the role of laccases remains largely unknown but their involvement in different steps of phenylpropanoid metabolism has been suggested. Fang et al. (2015) identified a vacuolar located ADE/LAC protein (anthocyanin degradation enzyme/laccase) responsible for the epicatechin-mediated anthocyanin degradation in litchi fruit during pericarp browning. Likewise, a gene encoding a putative rice laccase plays an important role in responses to abiotic stress (Cho et al., 2014). Our work strengthens the idea that the decrease in berry flavonoid content induced by HT involves an enzymatic-mediated degradation of these molecules in the vacuolar compartment. Indeed, *laccases, polyphenol oxidase, class III peroxidase*, and β*-glucosidase* genes were deregulated in local HT conditions (Supplementary Table 12). Particularly, the role of vacuolar class III peroxidase in anthocyanin degradation has been highlighted in different plant species including grape (Calderon et al., 1992; Vaknin et al., 2005; Zipor and Oren-Shamir, 2013; Zipor et al., 2015). For instance, a transcriptional up-regulation of *VviPrx31* (VIT_14s0066g01850) was observed in berries exposed to HT (Movahed et al., 2016; and Supplementary Table 1). In Petunia, its overexpression reduced anthocyanin contents in petals exposed to heat.

Finally, genes involved in stilbenes and lignins biosynthesis were also specifically altered by HT during berry development. These alterations may modify berry texture and tolerance to the environment.

### Heat deregulation of green berry transcriptome can contribute to delay veraison

During the herbaceous stage, heating significantly affected categories associated with stress and secondary metabolism (as discussed above), as well as categories associated with transport, cell wall, or hormones (Figure 5; Supplementary Table 15). The deregulation of these categories could explain, at least in part, the delayed veraison typified by the late accumulation of sugars and anthocyanins in GHT clusters (Figure 2).

#### Membrane transporters

Many genes encoding proteins related to transport processes were enriched in GHT conditions (Supplementary Table 15). These genes were mainly repressed in GHT berries, in agreement with previous reports indicating that HT exposure led to a decrease in micronutrient transport. Genes involved in the calcium homeostasis were also downregulated by applying HT to green berries, and particularly CNGCs genes. CNGCs are nonspecific cation channels that are regulated by cyclic nucleotides such as cAMP or cyclic GMP (Ward et al., 2009) and that contribute to Ca^2+^ signaling in the context of developmental processes, biotic and abiotic stress responses (Jha et al., 2016). Calcium may act through facilitating developmental and stress response signaling, stabilizing membranes, influencing water relations and modifying cell wall properties through cross-linking of de-esterified pectins (Hocking et al., 2016). Beside a sum of DEGs potentially linked to calcium homeostasis, the HT also impacted numerous genes related to signaling (protein kinase, transcription factors, Ca^2+^-binding proteins) (Supplementary Table 1). Potassium transporter gene expression was also altered after GHT exposure. As potassium is required for cell expansion, alteration in the expression of its transporters may affect berry growth. Additionally, as K^+^ transporters and channels are known targets of ABA (that play an important role in berry ripening) (Davies et al., 2006), the alteration of their expression during the herbaceous stage may contribute to delay fruit ripening. MATE efflux transporters and ABC transporters were specifically affected by HT. For instance, while members of subfamily B were up-regulated at GHT, MATE, and ABCC and G were down-regulated. Interestingly, ABCG, the most affected subfamily, is involved in fruit maturation and exhibits a protective role (detoxification, vacuolar transport of ABA and glucosyl ester, anion transport) (Andolfo et al., 2015). It is the largest plant ABC transporter subfamily divided in two groups: WBCs and PDRs. The first group is involved in the extrusion of cuticular lipids and the second, in resistance to pathogens, antimicrobial terpenoids and auxinic herbicides, and in transport of signaling molecules or in secretion of volatile compounds (Kretzschmar et al., 2011; Andolfo et al., 2015). The repression of most of these transporters could potentially contribute to decrease anthocyanin accumulation.

The down-regulation of sugar transporter genes by HT fits well with earlier reports showing that HT results in the delay or arrest of the ripening process and of the accumulation of sugars (Greer and Weston, 2010; Greer and Weedon, 2013). However, in our experimental conditions, the postponing of the ripening process appears only when HT is directly applied on green clusters (GHT). At later stages, most of these sugar transporters were either down-regulated or not affected by HT (Supplementary Table 15). Altogether, these modifications in the amount of transporter transcripts may affect the berry physiology by modifying the pH and the nutrient content. As a consequence, amounts of certain primary and secondary metabolites were also affected in heated berries, resulting in the delayed onset of veraison.

#### Cell wall metabolism

Genes involved in cell wall composition were strongly altered in green berries during HT, highlighting the importance of cell wall adjustment (Supplementary Table 15). Heating effects on cell wall metabolism were also observed in VHT and RHT clusters. The fine-tuning of the cellulose-hemicellulose networks appears to be crucial for tolerance to heat (Tenhaken, 2014; Le Gall et al., 2015). The present transcriptomic data suggest differential cell wall synthesis and remodeling in heat-exposed berries, potentially affecting the overall fruit growth even if the exact consequences remain to be determined. In vineyard conditions, Dal Santo et al. (2013) found a correlation between the season climate and some differentially expressed genes encoding enzymes involved in cell wall structural modifications (especially *CESs, PAEs*, and *XETs*). XETs contribute to cell wall expansion and loosening, through their action on xyloglucan frame. After whole grape exposure to heat, an increased level of *XETs* transcripts was measured in warmed green berries. It was postulated that enhanced expression could be related to the adaptation of berry volume to temperature and the need for more flexible cell walls (Rienth et al., 2014). Our data reinforce these observations with 19 up-regulated *XETs* in GHT clusters. Cell wall loosening through increase level of expansin might help to maintain cellular functions during HT (Le Gall et al., 2015).

#### Hormone homeostasis and signaling

Fruit development and responses to environmental cues are controlled by plant hormones (Davies and Böttcher, 2009; Kuhn et al., 2014) and particularly by ABA. The present work revealed that berry heating significantly affects the expression of genes involved in ABA metabolism and signaling (Supplementary Table 9). The deregulation of ABA biosynthesis and signaling by applying HT during the herbaceous stage may contribute to the postponing of veraison and/or help to face HT. Interestingly, NCED expression and particularly *VviNCED2* and *VviNCED4* was impacted in GHT but also in VHT and RHT clusters. The down regulation of these 2 genes contrasts with data from Carbonell-Bejerano et al. (2013) reporting an HT-induction of these two genes after veraison and an increased ABA level in full ripe heated berries. The differences between both studies might be due to differences in the genotype and/or in the experimental conditions (temperature regime, microenvironment versus whole plant exposure, stress kinetic). Genes involved in ABA signaling were also affected. For instance, *VviABI3* (VIT_07s0005g05400) strongly accumulated in GHT berries (Supplementary Table 9). ABI3 is a B3-domain TF that is a part of the core ABA signaling network and the corresponding gene is up-regulated in Cabernet Sauvignon berries treated with ABA (Rattanakon et al., 2016). In normal growth conditions, *VviABI3* transcripts accumulated during the lag phase prior veraison in Cabernet Sauvignon (Deluc et al., 2007). Thus, heating seems to accelerate the expression of *VviABI3* during the herbaceous stage. In Arabidopsis, *ABI3* is exclusively expressed in seeds, controlling gene expression programs that are essential to achieve seed maturation (Baud et al., 2016). Whereas the role of ABI3 in nonseed tissues remains unknown, a functional connection was demonstrated between ABI3 and HSFs in sunflowers embryos and during seed development in Arabidopsis (Rojas et al., 1999; Kotak et al., 2007b). Rattanakon et al. (2016) identified others TFs showing modified expression level after ABA treatment of grape organs, these TFs belonging to the AP2/ERF, NAC, bZIP/ABRE, and MYC/MYB families. In berries exposed to heat, more than 20 transcripts from this set of TFs were deregulated what can be potentially due to ABA action (Supplementary Table 15). The strongest deregulated TF of this list corresponds to the bZIP/ABRE *Abscisic Acid Insensitive protein 5* (*VviABI5*; VIT_06s0080g00340, VIT_08s0007g03420) that significantly accumulated in GHT clusters whatever the treatment duration (Supplementary Table 15). ABI5 is depicted as a key regulator in the ABA signaling pathway, and a recent work highlighted its contribution in the ABA-dependent stimulation of plant thermotolerance (Lee et al., 2015). After interaction with the plant-specific RNA-binding protein FCA, ABI5 enhanced antioxidant activity under HT conditions. Particularly, ABI5 promotes the expression of genes encoding antioxidants, including *1-CYSTEINE PEROXIREDOXIN 1* (*PER1*). In agreement with these data, the heat-induced expression profiles of *VviABI5* and *VviPER1* (VIT_05s0020g00600) were closely related in berries, suggesting a conserved mechanism in the fruit (Supplementary Table 1). Peroxiredoxins, which are thiol-based peroxidases, enhance plant tolerance to oxidative and heat stresses. More broadly, in this work, 70 DEGs were identified in heated berries as ROS (reactive oxygen species) scavenging/detoxifying enzymes and various antioxidants (Supplementary Table 1). This kind of adaptative response, described in different plants exposed to HT (de Pinto et al., 2015), may help the berries to manage potential damages due to oxidative burst. Indeed, ROS mainly attributable to NADPH oxidase activity accumulate under stress conditions including HT (Miller et al., 2009). Accordingly, two *VviRBOH* (*respiratory burst oxidase homolog*; VIT_14s0060g02320, VIT_01s0150g00440) genes were up-regulated in heat-exposed berries (Supplementary Table 1).

Besides ABA, our transcriptomic data (Supplementary Table 1) also suggest that HT affects the metabolism of auxin, ethylene and jasmonic acid. These hormones are involved in the control of grape berry development (Davies and Böttcher, 2009) and in the activation of key genes responsible for HT response (Bokszczanin and Fragkostefanakis, 2013; Sharma and Laxmi, 2015). For instance, indole-3-acetic acid (IAA) inhibits berry growth, sugar and anthocyanin accumulation (Kuhn et al., 2014) and prevents ripening. Indeed, the decrease in IAA content and the increase of its conjugated form are needed to induce ripening (Davies et al., 1997; Böttcher et al., 2010). Interestingly, our work showed that application of a HT to green berries resulted in the reduction of *IAA-amido synthetase* (*VviGH3*, VIT_07s0005g00090) transcript abundance and in an increase in the expression of two transcripts encoding IAA-amino acid hydrolases (VIT_11s0016g02700, VIT_08s0007g02740), thus suggesting that heating is disrupting IAA conjugating process in GHT clusters and therefore postponing the onset of ripening. Further work is needed to address the respective role of each hormone and their interactions in the context of grape berry grown under HT.

## Conclusions

This work provides the first molecular data describing the effect of HT at the microclimate level and brings important information on the consequences of temperature elevation in the context of leaf removal practice. HT effects depend both on the developmental stage and on the stress duration. Heating delayed the onset of veraison and strongly altered the berry biochemical composition at harvest. These physiological modifications could be partly explained by the deep remodeling of heated berry transcriptome. The intrinsic capacity of grape berries to perceive heat stress and to build adaptive responses is suggested by the deregulation of categories such as “stress responses,” “protein metabolism” and “secondary metabolism.” Additionally, important changes in processes related to “transport,” “hormone” and “cell wall” might contribute to the postponing of veraison. Furthermore, opposite effects depending on heat duration were observed for genes encoding enzymes of the general phenylpropanoid pathway, suggesting that the HT-induced decrease in anthocyanin content may result from a combination of transcript abundance and product degradation. However, one cannot exclude that this process could also be regulated at the protein level as HT strongly affects protein homeostasis related genes. Finally, the data reported here provide a rich transcriptomic resource for functional characterization of the genes that potentially control HT response and/or adaptation in grapevine. The functional characterization of some putative candidates is in progress.

## Author contributions

DL and PP designed the research; DL oversaw the research; JP, PP, and DL performed the greenhouse experiments; FL, JC, and DL carried out the RNA extraction for transcriptomic analysis and performed RT-QPCR; CK performed the bioinformatics analysis; JC, JP, GH, and CR did the metabolic analysis; FL, CK, and DL analyzed and interpreted the data; FL, CK, SD, and DL drafted the manuscript; PP and EG critically revised the manuscript. All authors read and approved the final manuscript.

## Funding

This research received funding from the Agence Nationale de la Recherche for the project “DURAVITIS” (grant no. ANR-2010-GENM-004-01).

### Conflict of interest statement

The authors declare that the research was conducted in the absence of any commercial or financial relationships that could be construed as a potential conflict of interest.
