# Supplementary material for: Dissecting the Biochemical and Transcriptomic Effects of a Locally Applied Heat Treatment on Developing Cabernet Sauvignon Grape Berries
Source: Front Plant Sci. 2017 Jan 31;8:53. doi: 10.3389/fpls.2017.00053 (PMC5281624; doi:10.3389/fpls.2017.00053)
Supplement: Supplementary file 16 [file Image1.PDF]

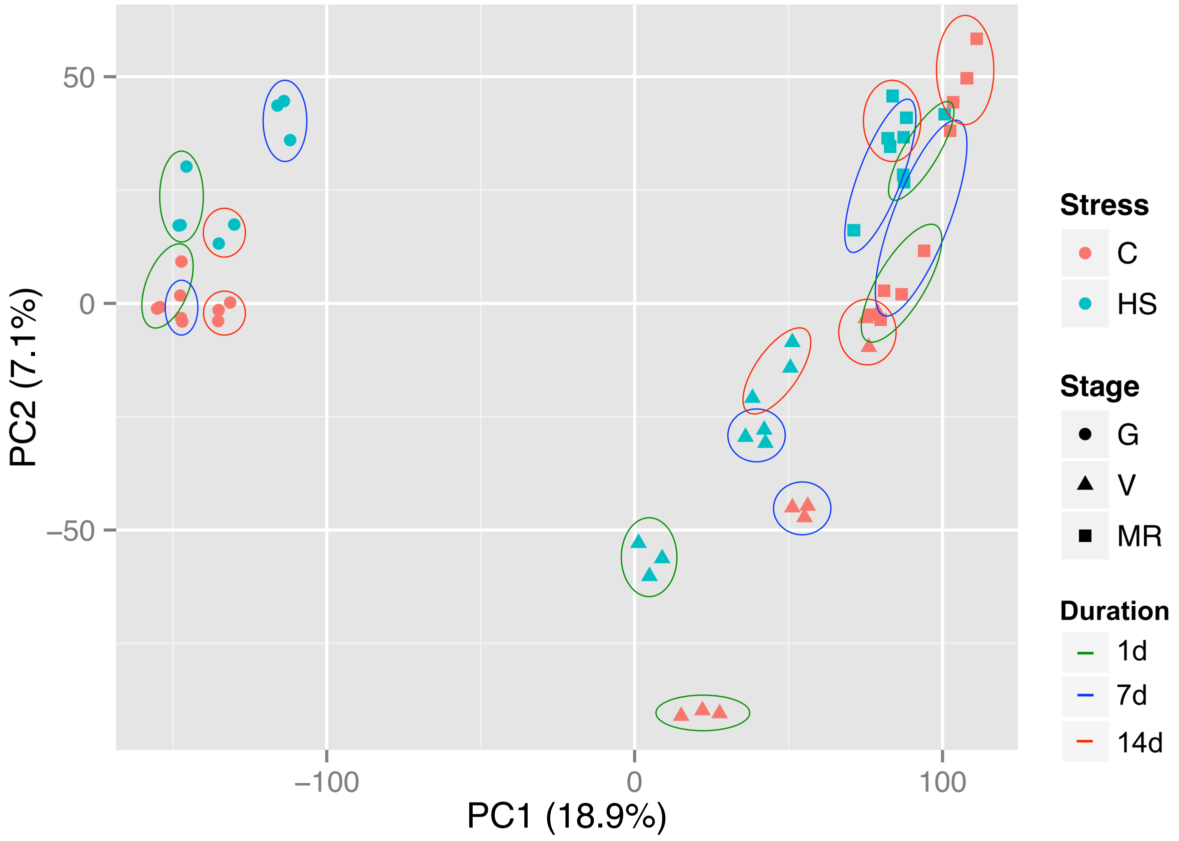

Supplementary Figure 1: Principal component analysis of the whole normalized gene expression dataset. Red and blue symbols represent control and heated samples, respectively. Solid lines encircle the three replicates of each stage subjected to the same treatment, and the different colour lines represent the treatment duration (1d, 7d or 14d). G: green; V: véraison; MR: middle ripening
