# Supplementary material for: Dissecting the Biochemical and Transcriptomic Effects of a Locally Applied Heat Treatment on Developing Cabernet Sauvignon Grape Berries
Source: Front Plant Sci. 2017 Jan 31;8:53. doi: 10.3389/fpls.2017.00053 (PMC5281624; doi:10.3389/fpls.2017.00053)
Supplement: Supplementary file 17 [file Image2.PDF]

A

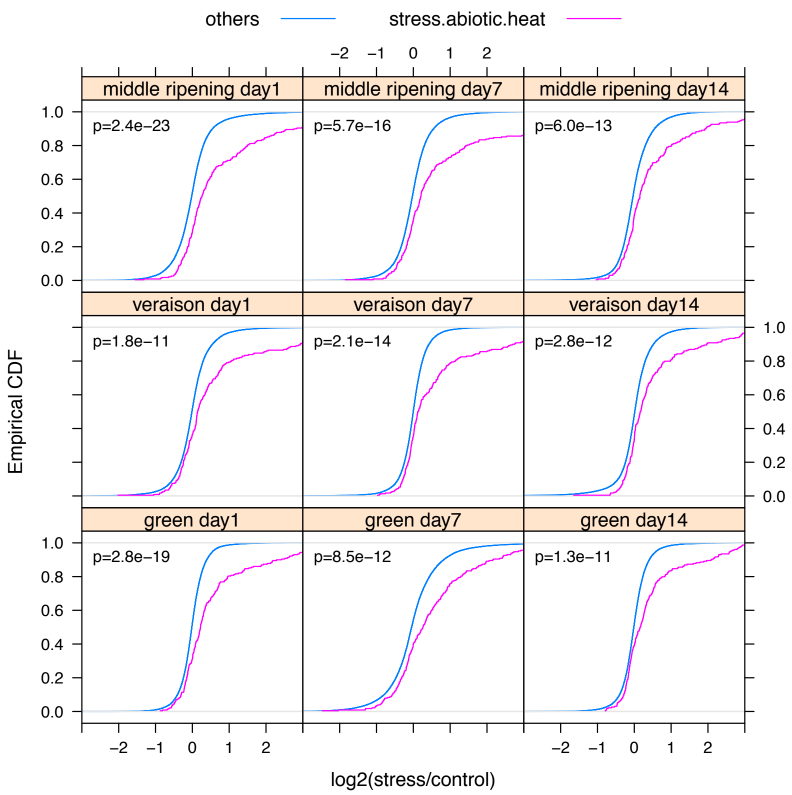

B

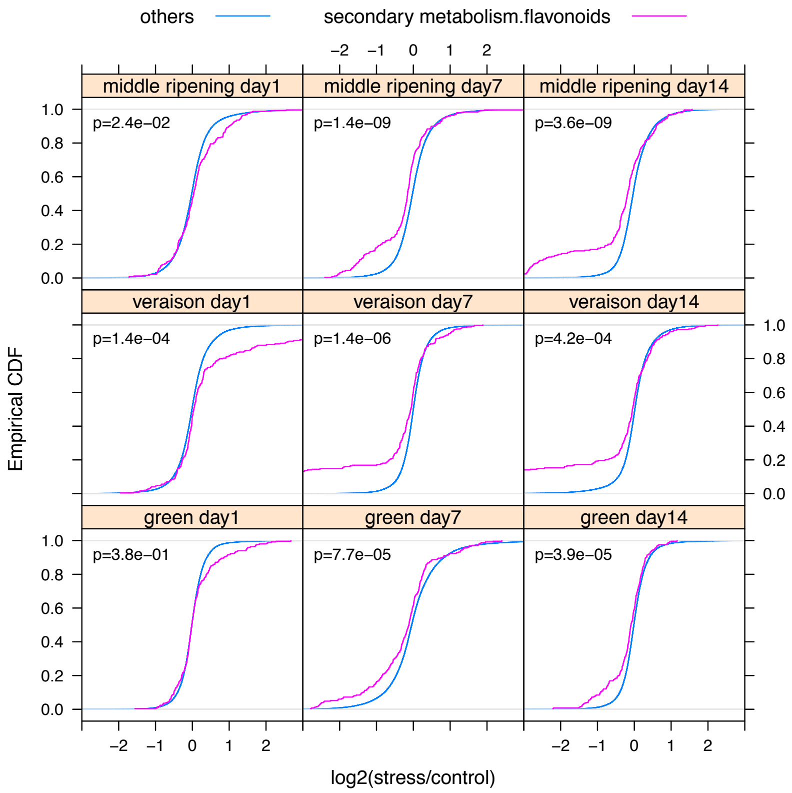

Supplementary Figure 2. Empirical cumulative distribution function (ECDF) plots illustrating the differential expression between treatment and control for all genes belonging to a given MapMan category (purple) compared to all other genes (cyan). Heat stress (A), Flavonoid secondary metabolisms (B). One panel for each developmental stage and treatment timepoint. Purple lines shifted to the right roughly indicate a tendency for the genes of the category towards over-expression, purple lines shifted to the left a tendency towards under-expression. P-values are based on a Wilcoxon Rank-Sum test.
