# Supplementary material for: Dissecting the Biochemical and Transcriptomic Effects of a Locally Applied Heat Treatment on Developing Cabernet Sauvignon Grape Berries
Source: Front Plant Sci. 2017 Jan 31;8:53. doi: 10.3389/fpls.2017.00053 (PMC5281624; doi:10.3389/fpls.2017.00053)
Supplement: Supplementary file 18 [file Image3.PDF]

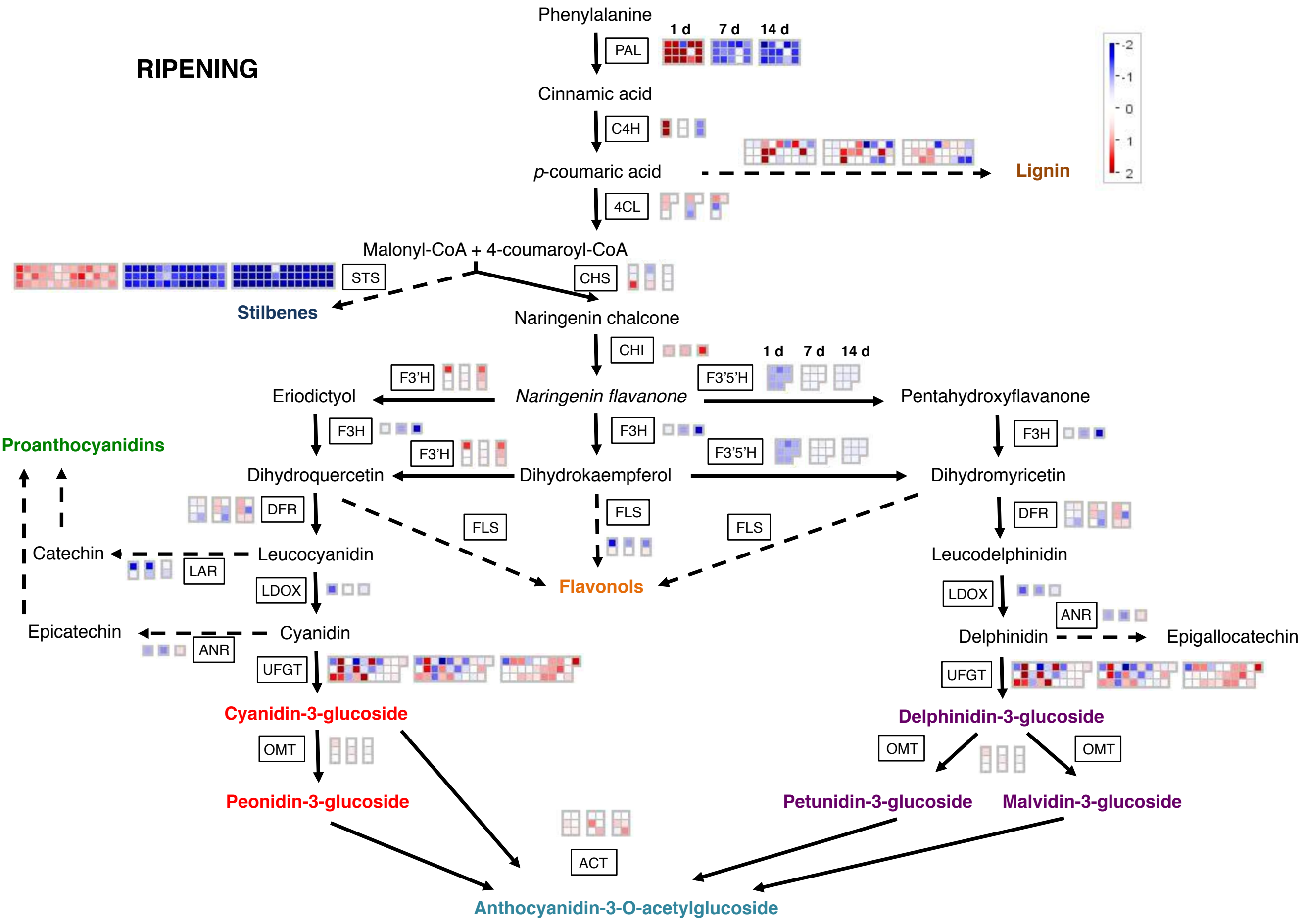

Supplementary Figure 3. MapMan visualisation of heat differentially expressed genes associated to the phenylpropanoid pathway in ripening berries. Each box corresponds to a treatment duration (1, 7 and 14 days). The expression ratio of each gene (log2 HT/Control) is represented by a coloured square following the color scale. Red : expression up-regulated under HT; blue : expression down-regulated under HT; white : no significant differential expression.
